# Exercise and Weekly Sirolimus (Rapamycin) in Older Adults: RAPA‐EX‐01 Randomised, Double‐Blind, Placebo‐Controlled Trial

**DOI:** 10.1002/jcsm.70274

**Published:** 2026-04-15

**Authors:** Brad Stanfield, Brian Leroux, Matt Kaeberlein, Julie Jones, Ruth Lucas

**Affiliations:** ^1^ Royal New Zealand College of General Practitioners Wellington New Zealand; ^2^ University of Auckland Auckland New Zealand; ^3^ University of Washington Seattle Washington USA; ^4^ Optispan Inc. Seattle Washington USA; ^5^ BioValeo Wellington New Zealand

**Keywords:** aging, exercise training, Geroscience, mTOR, mTORC1 inhibition, physical function, randomised controlled trial, rapamycin, sarcopenia, sirolimus

## Abstract

**Background:**

Preclinical models suggest alternating activation and inhibition of mechanistic target of rapamycin complex 1 (mTORC1) could enhance adaptation to exercise (‘cycling hypothesis’). Whether this concept translates to older adults is unknown. This exploratory trial assessed whether once‐weekly sirolimus (rapamycin) 6 mg enhances or inhibits functional gains from a home‐based exercise programme.

**Methods:**

In this randomised, double‐blind, placebo‐controlled trial, 40 sedentary adults aged 65–85 years (mean 72.2 years; 47.5% female) were assigned (1:1) to sirolimus (rapamycin) 6 mg or matched placebo once weekly for 13 weeks. Both groups performed a standardised home‐based resistance (chair‐stands) and endurance (exercycle) programme three times/week. The primary outcome was the change in 30‐s chair‐stand repetitions at 13 weeks (intention‐to‐treat; ANCOVA adjusted for baseline performance, age stratum and sex). Complete‐case (CC) and per‐protocol (PP) analyses were prespecified sensitivity analyses. Secondary outcomes included grip strength, 6‐min walk distance, SF‐36 physical and mental component scores, C‐reactive protein and several epigenetic age measures. Safety was assessed through adverse‐event monitoring and laboratory tests.

**Results:**

Both groups improved chair‐stand performance. The primary intention‐to‐treat analysis showed an adjusted mean difference (sirolimus–placebo) of −2.13 repetitions (95% CI −4.61 to 0.34; *p* = 0.089). Sensitivity analyses favoured placebo and reached statistical significance: complete‐case analysis (16 sirolimus, 19 placebo) showed a difference of −2.46 repetitions (95% CI −4.87 to −0.06; *p* = 0.045) and per‐protocol analysis (15 sirolimus, 16 placebo) showed −3.44 repetitions (95% CI −5.86 to −0.99; *p* = 0.007). Secondary functional outcomes also favoured placebo but were not statistically significant: the adjusted mean difference for 6MWD was −4.87 m (95% CI −28.97 to 19.71; *p* = 0.706) and for grip strength was −1.13 kg (95% CI −3.52 to 1.18; *p* = 0.344). SF‐36 scores showed small, non‐significant differences favouring placebo. Quality‐of‐life scores showed small, non‐significant differences favouring placebo. Seventeen participants (85%) in each arm reported ≥ 1 adverse event, but the total burden was higher with sirolimus (99 vs. 63 events), including one possibly drug‐related serious adverse event (pneumonia).

**Conclusion:**

In this exploratory trial, once‐weekly sirolimus (rapamycin) 6 mg did not enhance, and in sensitivity analyses, it may have modestly attenuated short‐term functional improvements from a home exercise programme in older adults. The regimen also increased the burden of minor adverse events and may have contributed to one serious infection. Future trials with longer treatment duration or less frequent/lower dosing are needed to determine whether a favourable benefit–risk profile can be achieved.

**Trial Registration:** ACTRN12624000790549

## 1 Background and Rationale

Loss of muscle mass and strength accelerates after midlife, contributing to falls, disability, hospital admission and mortality [1, 2]. Exercise and adequate protein intake can mitigate these declines, yet some older adults show limited anabolic responses [2].

The mechanistic target of rapamycin complex 1 (mTORC1) is a central regulator of skeletal muscle protein synthesis and hypertrophy and is implicated in age related anabolic resistance [3]. Overload and growth factor studies in rodents established that intact mTORC1 signalling is required for skeletal muscle hypertrophy: pharmacologic mTORC1 inhibition with rapamycin largely prevents load‐induced compensatory hypertrophy in rodents by inhibiting the IGF‐1/PI3K/Akt/mTOR pathway [4, 5, 6]. A single large dose of rapamycin also abolishes the post‐exercise rise in muscle protein synthesis in young men [7].

More recent preclinical data, however, are conflicting. Although high‐dose daily doses reliably blunt training adaptations, low‐dose or intermittent rapamycin dosing regimens often spare and occasionally enhance functional outcomes in aged mice [8, 9]. Partial mTORC1 inhibition reverses sarcopenia in rodents by increasing muscle mass and fibre cross‐sectional area while downregulating senescence and denervation gene programmes [10]. With aging, basal mTORC1 activity appears chronically elevated in some tissues, potentially suppressing autophagy and accelerating atrophy [3, 11]. Taken together, these data indicate that dose, schedule and timing relative to exercise are critical determinants of whether mTORC1 inhibition impairs, spares or potentially augments adaptation.

This tension has motivated the ‘cycling’ hypothesis: deliberately alternating mTORC1 activation (during and immediately after exercise) and inhibition (on non‐training days) might preserve or amplify training‐induced gains while still permitting autophagy‐mediated rejuvenation.

Sirolimus is a specific allosteric inhibitor of mTORC1, with partial inhibition of mTORC2 only after prolonged exposure [12]. In rodents, it extends lifespan and delays age‐related declines in cognition, kidney function and tendon function; transient midlife treatment reverses declines in the heart, immune system and oral cavity [13, 14, 15, 16, 17, 18, 19, 20, 21, 22, 23, 24, 25, 26, 27]. Based on prior weekly dosing studies in healthy adults and older cohorts, a 6 mg once weekly dose was expected to yield partial mTORC1 inhibition for several days each week without mTORC2 blockade [28]. Although we did not measure pharmacodynamic markers in this trial, prior human studies confirm that 5–6 mg weekly sirolimus inhibits S6K1 phosphorylation for ≥ 5–7 days, whereas mTORC2 remains unaffected [29].

Early human studies show that intermittent sirolimus (rapamycin) dosing is feasible and well tolerated in older adults. Small trials of daily dosing in coronary artery disease and aged volunteers reported minimal adverse effects but were underpowered for strength outcomes [30, 31]. Selective mTORC1 inhibition enhanced influenza vaccine responses and reduced infections over 12 months [29]. Weekly dosing in healthy men partially inhibited mTORC1 while improving insulin‐stimulated glucose uptake, and a larger study in healthy adults found that low‐dose weekly rapamycin was safe, increased lean tissue mass and reduced pain without adverse metabolic or haematologic changes [28, 32]. However, no published trial has combined once weekly sirolimus (rapamycin) with at‐home exercise while measuring validated functional endpoints such as the 30‐s chair‐stand test (30CST), 6‐min walk test or hand‐grip strength. The optimal cadence that balances autophagy activation and anabolic adaptation therefore remains unknown.

Beyond skeletal muscle, mTORC1 inhibition is hypothesised to slow systemic aging and reduce chronic inflammation (inflammaging). Therefore, determining whether intermittent dosing suppresses inflammatory markers (such as C‐reactive protein [CRP]) or slows biological aging clocks (DNA methylation) is critical for establishing the broader therapeutic potential of this regimen.

The RAPA‐EX‐01 trial was therefore designed as the first double‐blind, randomised, placebo‐controlled, exploratory trial to directly test the cycling hypothesis. We administered sirolimus (rapamycin) 6 mg (or placebo) once weekly, deliberately timed 24 h after the last exercise session of each week so as not to overlap with peak post‐exercise anabolic signalling, during a 13‐week progressive home‐based strength and endurance programme in sedentary 65‐ to 85‐year‐olds. The trial was primarily intended to generate precise effect‐size estimates and safety data to inform the design and sample size of a future confirmatory randomised controlled trial while also assessing whether the intervention produced a clinically meaningful signal of benefit, harm or neutrality.

## 2 Methods

### 2.1 Trial Design and Oversight

We conducted a single‐centre, randomised, double‐blind, placebo‐controlled, parallel‐group exploratory trial at Aotearoa Clinical Trials Trust (Auckland, New Zealand). The protocol was approved by the New Zealand Northern B Health and Disability Ethics Committee (2024 FULL 20084), registered with ANZCTR (ACTRN12624000790549) and published in *Trials* [33]. All participants provided written informed consent. The trial timeline and schedule of assessments are detailed in Table 1.

**TABLE 1:** Participant timeline and schedule of assessments.

Procedures and assessments	Visit 1	Visit 2	Visit 2a	Visit 3	Visit 4
	Screening	Baseline	Phone call	Interim assessment	EOS
	Day −28 to −1	Day 0	Within 6 days of baseline	Day 42 ± 7	Day 91 + 7
Informed consent	x				
Review of	x	x			
Subject					
Eligibility					
Demographics^a^	x				
Medical history	x				
Auscultate the heart to check for murmurs	x				
Vital signs^b^	x	x		x	x
ECG	x				
Haematology and biochemistry	x^c^			x^d^	x^c^
DNA methylation (TruDiagnostics)		x			x
SF‐36 Questionnaire		x			x
Assessment of ability to complete 30‐s chair‐stand test	x				
Randomisation		x			
Allocation of weekly		x		x	
Medication^e^					
Confirm participant Day 1			x		
Weekly participant contact			x^f^		
Adverse event collection		x	x	x	x
Distribute subject diary card		x		x	
Collect subject diary cards				x	x
30‐s chair‐stand test		x		x	x
Hand grip strength		x		x	x
6‐min walk test		x			x

^a^ Race, gender at birth, contact details, height and weight.

^b^ Resting heart rate, blood pressure and oxygen saturations.

^c^ Full blood count, eGFR, urea and electrolytes, LFTs, HbA1c, lipids, IGF‐1, CRP.

^d^ Full blood count, LFTs, lipids.

^e^ Allocation of investigational product for 6‐week period, followed by a 7‐week period.

^f^ Participants are to be contacted by phone each week. Participants who cannot be reached following two phone contact attempts in a week can be sent a text message or email.

### 2.2 Participants

We recruited community‐dwelling adults aged 65–85 years. To target a population likely to benefit from exercise initiation, inclusion required a sedentary lifestyle, defined as performing moderate intensity exercise for less than 15 min, three times per week. Exclusion criteria included uncontrolled diabetes (HbA1c ≥ 60 mmol/mol), significant renal (eGFR < 30 mL/min) or hepatic impairment, chronic corticosteroid use or contraindications to sirolimus. Participants taking rate‐limiting cardiac medications (e.g., beta‐blockers) were not excluded provided the condition was stable.

### 2.3 Randomisation and Blinding

Participants were randomly assigned (1:1) to sirolimus (rapamycin) or placebo using a computer‐generated sequence stratified by age (65–74 and 75–85 years). The allocation sequence was generated by an independent statistician and implemented via a secure REDCap module; allocation remained concealed from participants, care providers and outcome assessors throughout the trial.

### 2.4 Study Drug

Participants self‐administered three #000 capsules containing either 6 mg sirolimus (rapamycin; 3 × 2 mg Rapamune tablets) or matched microcrystalline cellulose placebo once weekly for 13 weeks. To minimise interference with exercise‐induced anabolic signalling, dosing was prescribed for ‘Day 6’ of each training week, approximately 24 h after the final exercise session.

### 2.5 Exercise Programme

Both groups performed an identical home‐based exercise programme three times per week (Days 1, 3, and 5) as outlined in Table 2. The regimen consisted of two components. First, a resistance component (30‐s chair stand) where participants performed maximum repetitions of standing from a seated position in 30 s. Although external weight was not added, progressive overload was achieved via ‘density training’, where participants were instructed to increase the number of repetitions performed within the fixed time interval as their fitness improved. Second, an endurance component using a provided magnetic resistance exercycle. The protocol followed a fixed 13‐week progression (Table 3), starting at 10 min (Level 1 resistance) and titrating up to 25 min (Level 5 resistance) by Week 9. Intensity was prescribed via mechanical targets (Resistance Level 1–5 and RPM 70–80) rather than heart‐rate zones. This ensured that participants on beta‐blockers or with chronotropic incompetence received a consistent physiological work stimulus. Where participants were unable to complete the exercycle training programme due to difficulty, the programme could be adjusted whereby the resistance setting was lowered, followed by a reduction in speed. If a participant still could not complete the training programme, they aimed to ride for as long as they were able before moving to the cooldown phase. Participants recorded exercise activities in their Participant Diary, and specifically for the exercycle sessions, the participants included the length of training, the RPM and the resistance setting.

**TABLE 2:** Weekly schedule.

Day 1	Training programme
Day 2	Rest day
Day 3	Training programme
Day 4	Rest
Day 5	Training programme
Day 6	Rest day
Day 7	Rest day

**TABLE 3:** Exercycle protocol.

Week	Warm‐up	Training	Cooldown
1	2 min@50 RPM	10 min@70–80 RPM, Resistance Level 1	2 min@45 RPM
2	2 min@50 RPM	15 min@70–80 RPM, Resistance Level 1	2 min@45 RPM
3	2 min@50 RPM	20 min@70–80 RPM, Resistance Level 2	2 min@45 RPM
4	2 min@50 RPM	25 min@70–80 RPM, Resistance Level 2	2 min@45 RPM
5	2 min@50 RPM	25 min@70–80 RPM, Resistance Level 3	2 min@45 RPM
6	2 min@50 RPM	25 min@70–80 RPM, Resistance Level 3	2 min@45 RPM
7	2 min@50 RPM	25 min@70–80 RPM, Resistance Level 4	2 min@45 RPM
8	2 min@50 RPM	25 min@70–80 RPM, Resistance Level 4	2 min@45 RPM
9	2 min@50 RPM	25 min@70–80 RPM, Resistance Level 5	2 min@45 RPM
10	2 min@50 RPM	25 min@70–80 RPM, Resistance Level 5	2 min@45 RPM
11	2 min@50 RPM	25 min@70–80 RPM, Resistance Level 5	2 min@45 RPM
12	2 min@50 RPM	25 min@70–80 RPM, Resistance Level 5	2 min@45 RPM
13	2 min@50 RPM	25 min@70–80 RPM, Resistance Level 5	2 min@45 RPM

### 2.6 Outcomes

The primary outcome was the change in lower‐body functional performance measured by the 30CST from baseline to Week 13. Secondary functional outcomes included the change in 6‐min walk distance (6MWD) assessed on a 30‐m indoor course and dominant‐hand grip strength measured as the maximum of three trials using a hydraulic dynamometer. Health‐related quality of life was assessed using the SF‐36 Physical and Mental Component Summary scores. Exploratory mechanistic endpoints included CRP, which serves as a marker of inflammation, and epigenetic age acceleration derived from genome‐wide DNA methylation profiling (TruDiagnostic TruAge). Safety was assessed via the incidence of adverse events (AEs; CTCAE v5.0) and changes in safety laboratory parameters.

### 2.7 Sample Size

The sample size of 40 participants (20 per arm) was determined to provide reasonable precision for estimating effect sizes and variance components in an exploratory setting [34, 35]. This sample size yields approximately 80% power at a two‐sided alpha of 0.05 to detect a large effect size (Cohen's *d* ≈0.80–0.90) on the primary outcome using ANCOVA adjusted for baseline values. Additionally, this sample size enables detection of AE rate ratios between approximately 2.4 and 3.7, providing reasonable sensitivity to clinically important safety signals. The target of 40 participants also represented the maximum feasible enrolment given single‐site operational capacity and funding constraints. No interim analyses or stopping rules were planned.

### 2.8 Statistical Analysis

All analyses were performed in R Version 4.4.0 and the R script is available as a supplementary file. The primary efficacy analysis was performed on an intention‐to‐treat (ITT) basis including all randomised participants. The Week 13 chair‐stand count was compared between arms using a linear regression model adjusted for sex, age group (< 75 vs. ≥ 75 years) and baseline values of chair‐stand, hand‐grip strength and 6MWD. Because heteroskedasticity was anticipated, standard errors and confidence intervals were estimated using a Rademacher wild bootstrap with 999 replicates. Missing outcome data were handled via multiple imputation using predictive mean matching (15 imputed datasets).

Secondary outcomes (grip strength, 6‐min walk, SF‐36 scores, CRP and epigenetic clocks) were analysed using the same baseline‐adjusted model and wild‐bootstrap approach. Prespecified sensitivity analyses included a complete‐case analysis (participants with paired baseline and Week 13 data) and a per‐protocol analysis (participants completing ≥ 75% of prescribed doses and exercise sessions). AEs were analysed using negative binomial regression to compare event counts between groups. As this was an exploratory trial, no adjustments were made for multiplicity.

## 3 Results

### 3.1 Recruitment and Baseline Data

**FIGURE 1:**
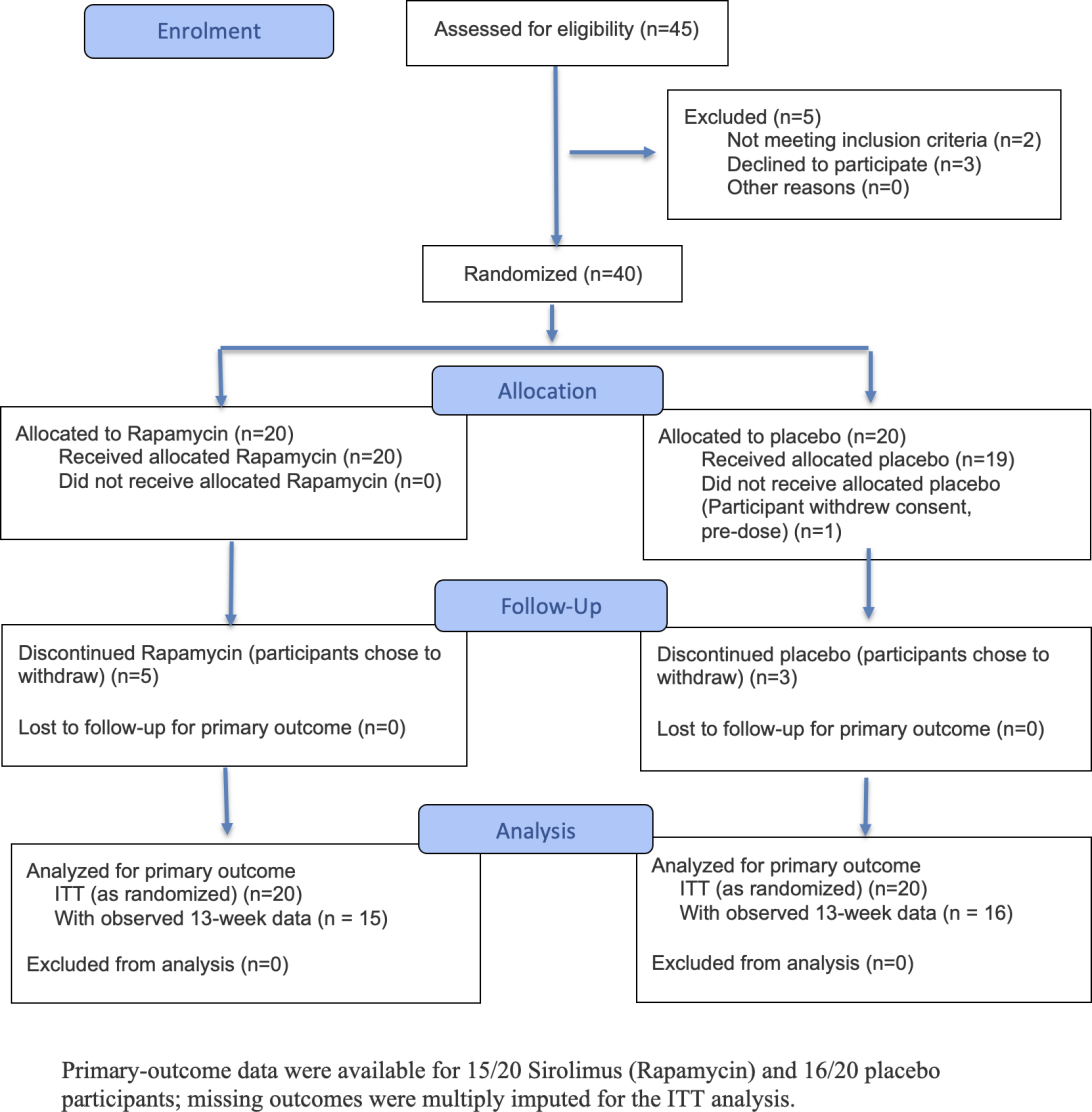
CONSORT flow diagram.

Between 29 July 2024 and 30 September 2024, 45 individuals were assessed for eligibility; 40 participants were randomised (20 assigned to sirolimus, 20 to placebo). Baseline demographic and clinical characteristics were well balanced between groups (Table 4). Detailed baseline vital signs, laboratory parameters and individual SF‐36 domain scores are provided as Table S1. Follow‐up was completed on 14 January 2025. Five participants in the sirolimus (rapamycin) arm and three in the placebo arm discontinued the intervention early, but their available data were included in the ITT analysis (Figure 1).

**TABLE 4:** Key baseline data.

	Placebo							Active							Overall						
	Mean	SD	Min	Max	Median	Q25	Q75	Mean	SD	Min	Max	Median	Q25	Q75	Mean	SD	Min	Max	Median	Q25	Q75
Female (proportion)	0.40							0.55							0.48						
Age (years)	71.95	5.49	65.00	82.00	71.50	66.75	75.75	72.40	5.06	65.00	81.00	71.50	68.75	76.50	72.18	5.22	65.00	82.00	71.50	67.75	76.50
Height (cm)	167.65	8.18	150.00	181.00	167.00	161.75	175.00	168.40	10.43	150.00	186.00	169.50	163.00	174.50	168.03	9.26	150.00	186.00	168.00	161.75	175.00
Weight (kg)	85.65	16.77	49.00	113.00	85.50	76.25	101.00	81.80	14.10	59.00	111.00	79.50	73.75	90.25	83.73	15.42	49.00	113.00	81.00	74.00	94.25
Chair‐stand test (reps)	14.30	3.42	9.00	22.00	14.00	12.50	17.00	13.75	2.12	10.00	17.00	13.00	12.00	16.00	14.03	2.82	9.00	22.00	13.50	12.00	16.00
Hand grip strength (kg)	34.05	8.58	24.00	53.00	32.00	26.75	39.25	30.75	9.00	21.00	50.00	29.00	22.75	34.75	32.40	8.84	21.00	53.00	31.00	25.75	37.25
6‐min walk distance (m)	506.30	63.53	347.00	624.00	526.00	454.75	542.00	494.45	55.70	408.00	607.00	489.50	456.25	531.25	500.38	59.27	347.00	624.00	503.00	454.75	540.25
CRP (mg/L)	2.15	1.57	1.00	5.00	1.00	1.00	3.00	2.10	2.59	1.00	10.00	1.00	1.00	2.00	2.09	2.06	1.00	10.00	1.00	1.00	3.00
Physical component summary (PCS)	49.92	4.39	40.60	57.36	50.39	47.33	52.50	48.94	5.68	37.54	57.48	49.64	46.58	52.40	49.49	4.74	37.54	57.48	50.00	47.27	52.06
Mental component summary (MCS)	51.96	4.61	35.47	56.74	52.70	50.98	54.37	49.52	7.89	23.30	57.74	51.24	47.69	54.88	50.66	6.12	23.30	57.74	51.42	50.00	54.23
PCGrimAge (years)	77.80	5.37	68.45	85.47	77.65	73.53	83.47	79.81	5.38	71.51	91.72	80.31	74.52	81.81	78.80	5.40	68.45	91.72	78.81	74.10	83.07
SystemsAge (years)	73.09	7.70	60.64	87.60	71.80	68.22	76.80	73.30	9.50	55.83	90.37	73.83	63.83	81.96	73.20	8.54	55.83	90.37	72.28	67.55	79.65
OMICmAge (years)	69.42	5.20	63.37	82.57	68.87	65.15	71.68	69.32	5.12	61.64	77.13	69.78	65.40	73.70	69.37	5.09	61.64	82.57	69.21	65.21	73.70
DunedinPACE (ratio)	1.06	0.14	0.83	1.40	1.08	0.96	1.12	1.03	0.12	0.88	1.34	0.99	0.95	1.08	1.05	0.13	0.83	1.40	1.03	0.96	1.10

### 3.2 Intervention Delivery and Adherence

Adherence to the protocol was high. Median medication adherence was 90.9% (IQR 80.8–100.0) in the placebo arm and 87.3% (IQR 76.6–100.0) in the sirolimus (rapamycin) arm. Exercise adherence was similarly robust, with mean session completion rates of 77.3% (SD 34.7%) and 72.6% (SD 38.1%), respectively (mean difference 4.7 percentage points; 95% CI −18.6 to 28.0; *p* = 0.69).

To address potential confounding by exercise intensity tolerance, we analysed daily session logs. Deviations from the prescribed workload (e.g., reducing resistance below the weekly target or stopping a session early due to fatigue) were rare, occurring in less than 1% of total scheduled sessions across both groups. Furthermore, the use of rate‐limiting cardiac medications (beta‐blockers) was balanced between arms (*n* = 2 in sirolimus; *n* = 2 in placebo), suggesting that chronotropic incompetence did not differentially affect the ability to achieve the prescribed mechanical workload targets.

### 3.3 Primary Outcome—30CST

**FIGURE 2:**
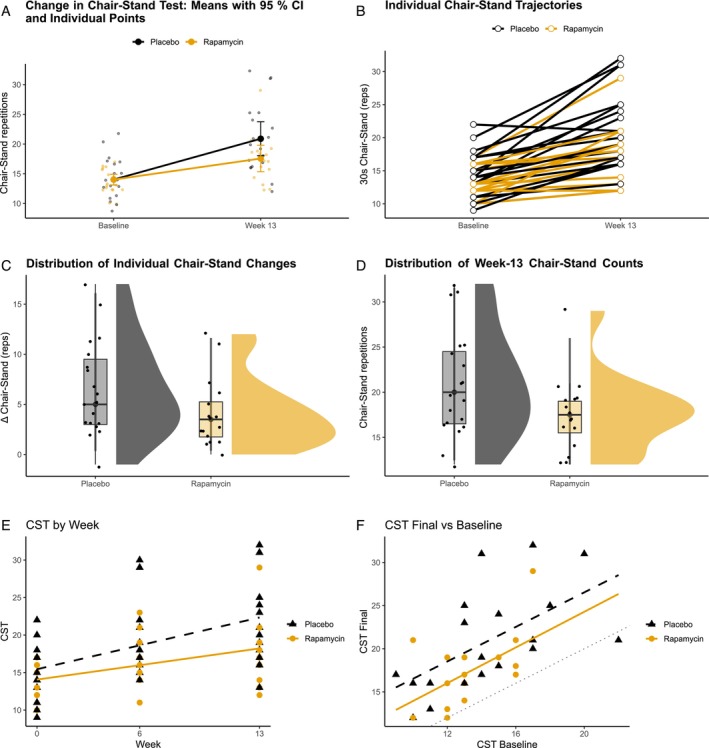
Panel (A) summarises group‐level performance at baseline and at 13 weeks. For each treatment group, the mean chair‐stand count is plotted, flanked by vertical bars representing approximate 95% confidence intervals. Individual participant values are drawn as faint points behind the means to convey the variability within each group. Panel (B) presents ‘spaghetti’ plots of individual trajectories from baseline to 13 weeks. Every participant is represented by a line; the treatment assignment is indicated by colour. This allows readers to see the distribution of responses rather than just the average change. Panel (C) depicts the distribution of change scores—13‐week count minus baseline count—for each group. Density estimates are combined with box plots and jittered points to illustrate both the shape and spread of the data. A narrow, right‐shifted distribution would imply consistent improvement; overlap between the groups suggests that differences may be modest. Panel (D) shows the distribution of absolute chair‐stand counts at 13 weeks for each group. As in Figure 2C, density plots and box plots are overlaid with individual data points. This panel focuses on end‐of‐study performance rather than change. Panel (E) plots chair‐stand counts at all available time points. The horizontal axis explicitly labels 0, 6 and 13 weeks, and a linear regression line is fitted within each treatment group. A steeper slope indicates a greater increase in repetitions over time. Panel (F) compares baseline and final chair‐stand counts for each participant. Group‐specific regression lines are fitted using all available data points and are extended across the full range of baseline values. A 45° reference line (grey dotted) indicates no change. Points above this reference line show improvement; points below show decline.

Both groups improved their lower‐body functional performance over 13 weeks (Figure 2A). In the primary ITT analysis (*n* = 40), the baseline‐adjusted mean difference in 30‐s chair‐stand repetitions at Week 13 (sirolimus minus placebo) was −2.13 repetitions (95% CI −4.61 to 0.34; *p* = 0.089). This represented a small‐to‐medium negative effect size (Cohen's *d* = −0.53).

Prespecified sensitivity analyses, which excluded non‐adherent participants or those with missing data, indicated a significant attenuation of benefit in the treatment arm. The complete‐case analysis (16 sirolimus, 19 placebo) yielded a mean difference of −2.46 repetitions (95% CI −4.87 to −0.06; *p* = 0.045; Cohen's *d* = −0.64). The per‐protocol analysis (15 sirolimus, 16 placebo) showed a large negative effect, with a difference of −3.44 repetitions (95% CI −5.86 to −0.99; *p* = 0.007; Cohen's *d* = −0.90).

### 3.4 Secondary Outcomes

Secondary functional and patient‐reported outcomes followed a similar pattern, with point estimates favouring placebo, though differences did not reach statistical significance.

The adjusted mean difference for the 6MWD was −4.87 m (95% CI −28.97 to 19.71; *p* = 0.706; Cohen's *d* = −0.26). Grip strength showed a difference of −1.19 kg (95% CI −3.52 to 1.18; *p* = 0.344; Cohen's *d* = −0.40).

Self‐reported health‐related quality of life (SF‐36) showed negligible between‐group differences. The adjusted mean difference for the Physical Component Summary was −2.76 points (95% CI −8.81 to 3.32; *p* = 0.376), and for the Mental Component Summary was −1.22 points (95% CI −4.16 to 1.91; *p* = 0.455).

Exploratory analysis of CRP showed a mean difference of +4.26 mg/L (95% CI −0.04 to 8.68; *p* = 0.152) in the sirolimus arm. However, this was driven by two outliers in the treatment group with marked elevations (17 and 50 mg/L) at Week 13; excluding these participants reduced the difference to < 1 mg/L. Epigenetic age measures showed mixed, non‐significant trends (Table 5).

**TABLE 5:** Primary and secondary outcome results.

Outcome	Baseline rapamycin mean ± SD	Baseline placebo mean ± SD	Week 13 rapamycin mean ± SD	Week 13 placebo mean ± SD	Adjusted difference ITT	95% CI ITT	*p* ITT	*t*‐statistic (df) (ITT)	*F*‐statistic (df1, df2) (ITT)	Partial eta^2^ (ITT)	Cohen's *d* (ITT)	Adjusted difference (CC)	95% CI (CC)
30‐s chair‐stand test (repetitions)	13.75 ± 2.12	14.30 ± 3.42	17.56 ± 4.18	20.89 ± 5.92	−2.13	−4.61 to 0.34	0.089	−1.65 (34)	2.73 (1, 34)	0.074	−0.526	−2.455	−4.87 to −0.06
Grip strength (kg)	30.75 ± 9.00	34.05 ± 8.58	31.71 ± 9.70	36.29 ± 9.50	−1.19	−3.52 to 1.18	0.344	−1.27 (34)	1.62 (1, 34)	0.045	−0.401	−1.5233	−4.00 to 1.03
6‐min walk (m)	494.45 ± 55.70	506.30 ± 63.53	529.62 ± 61.80	556.24 ± 64.75	−4.87	−28.97 to 19.71	0.706	−0.43 (34)	0.18 (1, 34)	0.005	−0.26	−3.8019	−27.76 to 20.71
SF 36 physical component summary	48.94 ± 5.68	49.92 ± 4.39	49.32 ± 10.57	55.22 ± 6.96	−2.76	−8.81 to 3.32	0.376	0.11 (33)	0.01 (1, 33)	0	−0.471	−1.2904	−7.32 to 4.38
SF 36 mental component summary	49.52 ± 7.89	51.96 ± 4.61	44.98 ± 6.27	43.75 ± 5.76	−1.22	−4.16 to 1.91	0.455	−0.73 (33)	0.53 (1, 33)	0.016	0.214	−0.5451	−3.33 to 2.19
C reactive protein (mg L^−1^)	2.10 ± 2.59	2.15 ± 1.57	5.63 ± 12.89	1.47 ± 0.62	4.26	−0.04 to 8.68	0.152	1.94 (38)	3.78 (1, 38)	0.09	0.516		
PC GrimAge clock (years)	79.81 ± 5.38	77.80 ± 5.37	78.15 ± 5.62	79.72 ± 5.69	−2.28	−4.93 to 0.44	0.098	−1.57 (33)	2.46 (1, 33)	0.069	−0.112		
SystemsAge clock (years)	73.30 ± 9.50	73.09 ± 7.70	73.57 ± 8.16	73.19 ± 8.14	1.04	−0.69 to 2.55	0.24	1.87 (33)	3.49 (1, 33)	0.096	0.137		
OMICmAge clock (years)	69.32 ± 5.12	69.42 ± 5.20	70.18 ± 4.43	70.32 ± 5.34	0.28	−0.74 to 1.40	0.592	0.22 (33)	0.05 (1, 33)	0.001	0.002		
DunedinPACE clock (unitless)	1.03 ± 0.12	1.06 ± 0.14	1.07 ± 0.11	1.07 ± 0.14	0.0035	−0.048 to 0.056	0.905	0.81 (33)	0.66 (1, 33)	0.019	0.04		

Outcome	*p* (CC)	*t*‐statistic (df) (CC)	*F*‐statistic (df1, df2) (CC)	Partial eta^2^ (CC)	Cohen's *d* (CC)	Adjusted difference (PP)	95% CI (PP)	*p* (PP)	*t*‐statistic (df) (PP)	*F*‐statistic (df1, df2) (PP)	Partial eta^2^ (PP)	Cohen's *d* (PP)
30‐s chair stand test (repetitions)	0.045	−1.70 (28)	2.89 (1, 28)	0.093	−0.64	−3.4382	−5.86 to −0.99	0.007	−2.25 (24)	5.05 (1, 24)	0.174	−0.901
Grip strength (kg)	0.219	−1.14 (28)	1.29 (1, 28)	0.044	−0.478	−2.2852	−4.78 to 0.25	0.079	−1.55 (24)	2.40 (1, 24)	0.091	−0.609
6‐min walk (m)	0.758	−0.25 (26)	0.06 (1, 26)	0.002	−0.42	−8.4113	−32.33 to 15.68	0.507	−0.48 (24)	0.23 (1, 24)	0.01	−0.514
SF 36 physical component summary	0.663	−0.39 (28)	0.15 (1, 28)	0.005	−0.668	0.501	−5.04 to 5.72	0.858	0.15 (23)	0.02 (1, 23)	0.001	−0.562
SF 36 mental component summary	0.7	−0.35 (28)	0.12 (1, 28)	0.004	0.205	−1.6619	−4.38 to 1.17	0.248	−0.95 (23)	0.91 (1, 23)	0.038	0.123
C reactive protein (mg L^−1^)												
PC GrimAge clock (years)												
SystemsAge clock (years)												
OMICmAge clock (years)												
DunedinPACE clock (unitless)												

Overall, across all secondary outcomes the confidence intervals spanned zero and the direction of effect usually favoured placebo, supporting the null hypothesis that once‐weekly sirolimus (rapamycin) does not improve and may modestly attenuate functional or quality‐of‐life gains from exercise over 13 weeks. Detailed descriptive statistics and adjusted estimates for all primary and secondary outcomes are presented in Table 5.

### 3.5 Safety and AEs

Safety was assessed in all 40 randomised participants. Seventeen participants (85%) in each arm reported at least one AE. However, the total burden of events was numerically higher in the sirolimus arm (99 total events vs. 63 in placebo; incidence rate ratio 1.57; 95% CI 0.86–2.87; *p* = 0.14). The majority of events were mild (CTCAE Grade 1), with headache, fatigue and upper respiratory tract symptoms being the most common (Table 6). Importantly, events adjudicated as possibly or probably related to the study drug were more frequent in the sirolimus arm (35% vs. 15%).

**TABLE 6:** Adverse events and laboratory summary.

Parameter	Rapamycin baseline	Rapamycin Week 13	Placebo baseline	Placebo Week 13	Effect (rapamycin vs. placebo)	95% CI	*p*
Participants	20	20	20	20			
Participants with ≥ 1 AE		17		17	0		
Participants with ≥ 1 SAE		1		0	1		
Total AEs		99		63	36		
Total SAEs		1		0	1		
Mean AEs per participant		4.95		3.15	1.8		
Rate ratio for AEs					1.57	0.86–2.87	0.14
Albumin (g/L)	38.9	36.3125	38.7	37.31578947	−0.99235291	−2.0767 to 0.049918	0.074304757
Alkaline phosphatase (U/L)	84.1	83.125	71.5	68.63157895	5.558858483	1.5334–10.049	0.012403952
ALT (U/L)	21.35	22.125	21.8	21.42105263	−0.261847698	−6.4278 to 6.2023	0.938379587
AST (U/L)	24.2	25.6	24.4	26.10526316	−0.845386164	−5.3749 to 3.6507	0.719028392
Basophils (10^9^/L)	0.09	0.06875	0.0825	0.076315789	−0.01492288	−0.042882 to 0.01286	0.302533476
Bilirubin (umol/L)	8.5	7.875	9.65	10	−1.205745805	−2.4368 to −0.0084656	0.051801228
Cholesterol/HDL ratio	3.145	3.3375	3.39	3.2	0.357053003	−0.01568 to 0.71116	0.060289733
Total cholesterol (mmol/L)	5.08	5.3	4.645	4.478947368	0.489984505	0.15821–0.81531	0.004137842
Creatinine (umol/L)	77.75	81.3125	84.7	88.42105263	−1.539563209	−6.5912 to 3.4041	0.544767618
eGFR (mL/min/1.73 m^2^)	76.15	72.625	72.8	70.36842	−0.33946	−4.96668 to 4.40918	0.943329247
Eosinophils (10^9^/L)	0.16	0.2	0.195	0.171052632	0.045189886	−0.017116 to 0.10965	0.153991273
GGT (U/L)	24.8	25.375	26.85	26.31579	−0.4353	−5.20371 to 4.30872	0.938002958
Globulin (g/L)	35.4	35.5625	36.55	34.84210526	1.340366342	−0.47622 to 3.0773	0.150854264
Haematocrit (ratio)	0.428	0.416875	0.416	0.411578947	−0.006282783	−0.018442 to 0.0057992	0.31364379
Haemoglobin (g/L)	140.85	136.375	140.2	137.7894737	−2.021025826	−5.0304 to 0.72381	0.192315257
HbA1c (mmol/mol)	40.5	42.3125	41.7	40.68421053	1.739615191	0.20142–3.2719	0.029612417
HDL cholesterol (mmol/L)	1.69	1.69125	1.435	1.460526316	−0.016016909	−0.17244 to 0.12957	0.837792368
CRP (mg/L)	2.1	5.625	2.15	1.473684211	4.279445345	−0.03773 to 8.6786	0.15008233
IGF 1 (ng/mL)	125.7894737	137.0625	118.65	116.4736842	12.98168867	−1.2555 to 27.422	0.074071986
LDL cholesterol (mmol/L)	2.84	2.9625	2.55	2.415789474	0.324863694	0.023128–0.61462	0.036232031
Lymphocytes (10^9^/L)	1.85	1.98125	1.805	1.884210526	0.096171202	−0.20825 to 0.41639	0.536648751
MCV (fL)	88.85	85.75	89.25	89.21052632	−2.896436792	−4.0334 to −1.6326	3.25624E‐06
Monocytes (10^9^/L)	0.55	0.5875	0.61	0.557894737	0.089299563	−0.019064 to 0.19953	0.131444507
Neutrophils (10^9^/L)	3.945	3.8875	4.22	4.257894737	0.022679619	−0.86641 to 0.8688	0.95953412
Platelets (10^9^/L)	261.25	261.625	264.3	254.9473684	17.6445312	1.5254–32.328	0.025145251
Potassium (mmol/L)	4.2	4.06875	4.125	4.173684211	−0.131406782	−0.2883 to 0.022563	0.110343363
Total protein (g/L)	74.3	71.875	75.25	72.15789474	0.108620925	−2.1328 to 2.1452	0.920371694
RBC (10^12^/L)	4.847	4.88625	4.673	4.628947368	0.062305731	−0.065747 to 0.18721	0.359506461
Sodium (mmol/L)	140.45	139.6875	140.3	140.3157895	−0.854594658	−1.9421 to 0.26075	0.139196836
Triglycerides (mmol/L)	1.34	1.60625	1.62	1.515789474	0.263522143	−0.1276 to 0.66205	0.205133489
Urea (mmol/L)	5.8	5.90625	6.575	6.631578947	−0.076925945	−0.76184 to 0.55728	0.821061955

One serious AE occurred in a participant in the sirolimus (rapamycin) arm (ID MDMR‐00015) who developed community‐acquired pneumonia with nasal congestion and severe constipation on 2 October 2024. The participant was hospitalised overnight and treated with intravenous antibiotics and steroids; constipation was managed conservatively. Symptoms resolved, but the participant withdrew from the trial. No other participants discontinued because of AEs. Of note, this participant had only received a single dose of sirolimus (rapamycin); however, given sirolimus (rapamycin)'s immunosuppressive properties, a causal contribution to this serious AE cannot be excluded.

### 3.6 Lab Safety Analysis

Analysis of safety laboratory parameters revealed several statistically significant but clinically modest shifts in the sirolimus arm (Table 6). Mean corpuscular volume was lower (adjusted difference −2.90 fL; *p* < 0.001), whereas platelet count (+17.6 × 10^9^/L; *p* = 0.025), alkaline phosphatase (+5.56 U/L; *p* = 0.012), LDL cholesterol (+0.32 mmol/L; *p* = 0.036) and HbA1c (+1.74 mmol/mol; *p* = 0.030) were slightly elevated compared with placebo. Bilirubin and albumin trended lower, whereas insulin‐like growth factor 1 (IGF‐1) trended higher, though these did not reach statistical significance. As noted in the secondary outcomes, CRP levels were driven by outliers and showed no consistent pattern.

## 4 Discussion

### 4.1 Principal Findings

This randomised, double‐blind, exploratory trial evaluated whether once‐weekly sirolimus (rapamycin) 6 mg, timed to avoid peak training windows, could enhance functional adaptations to a 13‐week resistance and endurance programme in sedentary older adults.

Contrary to the ‘cycling hypothesis’ derived from preclinical models, we found no evidence of benefit. Instead, sensitivity analyses (complete‐case and per‐protocol) revealed a statistically significant blunting of functional gains in the sirolimus arm. Specifically, participants taking the drug performed fewer chair‐stand repetitions and showed non‐significant trends toward reduced walking distance and grip strength compared with placebo. Safety monitoring identified a higher burden of minor AEs and one serious infection (pneumonia) in the treatment arm.

### 4.2 Context and Comparison With Previous Studies

This observation is consistent with classic rodent overload studies in which rapamycin largely prevented compensatory hypertrophy and with acute human data showing that a single large dose abolishes the post‐exercise rise in muscle protein synthesis [4, 5, 7]. This parallels findings from trials of metformin (an indirect mTORC1 inhibitor), where concurrent administration attenuated improvements in muscle mass and strength in older adults [36, 37].

Notably, very recent preclinical data in mice showed no blunting of exercise adaptations with rapamycin; differences in species, sex, dose or timing relative timing likely explain the apparent discrepancy [8].

Although our trial is the first to combine once weekly sirolimus (rapamycin) with exercise in older adults, its findings differ from those of the PEARL study, which administered rapamycin weekly without an exercise programme and reported modest improvements in female lean body mass and pain [28]. Differences in trial duration (48 weeks vs. 13 weeks), endpoints (body composition and symptoms versus functional performance) and the absence of an anabolic stimulus in PEARL likely account for these divergent results. Together, the evidence suggests sirolimus (rapamycin)'s effects are context dependent: possibly beneficial in sedentary settings over longer durations, but counterproductive when overlapping with short term exercise training.

### 4.3 Mechanistic Explanations

The failure to achieve a ‘best of both worlds’ effect (the concurrent realisation of sirolimus‐induced geroprotection and exercise‐induced muscle hypertrophy) likely stems from a pharmacokinetic mismatch. The ‘cycling hypothesis’ relies on a clear separation between the catabolic/autophagic phase (drug effect) and the anabolic phase (recovery from exercise). Although we dosed sirolimus 24 h after the final weekly exercise session, the drug's terminal half‐life of approximately 62 h implies that biologically active concentrations persisted well into the subsequent training week [38]. Consequently, mTORC1 (the master regulator of translation initiation) likely remained partially inhibited during the critical post‐exercise windows of the following sessions, thereby dampening the hypertrophic response.

### 4.4 Safety Implications

Although weekly sirolimus is often discussed as a potential longevity therapeutic, our data warrant caution regarding its short‐term safety profile in active older adults. The treatment arm experienced a 57% higher incidence rate of AEs, driven by minor infections and constitutional symptoms. The occurrence of community‐acquired pneumonia requiring hospitalisation in one participant, though singular, aligns with the drug's known immunosuppressive mechanism.

### 4.5 Strengths and Limitations

Strengths of this trial include the double‐blind, placebo‐controlled design, high adherence to the home‐based protocol, and the use of validated functional endpoints. We also addressed potential confounders such as beta‐blocker use and exercise intensity tolerance, finding no evidence that these factors biased the results.

A key limitation was the home‐based nature of the exercise intervention. Unlike gym‐based training with external weights, our chair‐stand protocol relied on body weight. Although we employed ‘density training’ (increasing repetition volume within a fixed time) to ensure progressive overload, this approach may have a lower ceiling for maximal strength development than heavy resistance training. Furthermore, the trial was limited to 13 weeks, so the longer‐term effects of combining sirolimus (rapamycin) with exercise, particularly with lower doses or less frequent administration, remain unknown. Finally, we did not perform muscle biopsies or pharmacokinetic monitoring, so our mechanistic attribution of the ‘blunting’ effect to persistent mTORC1 inhibition remains inferential.

Finally, although we did not use accelerometry to quantify total non‐exercise physical activity (NEPA), participants recorded additional voluntary physical activity (e.g., walking, gardening) in daily diaries; review of these records suggests no systematic difference in extra‐curricular activity between groups.

### 4.6 Implications for Future Research

Future research should explore regimens that lengthen the time between doses (e.g., every 3–6 weeks). Given sirolimus's ~62‐h terminal half‐life, this interval would allow for near‐complete drug clearance, ensuring mTORC1 signalling can recover to support exercise adaptations. Furthermore, a longer interdose interval may mitigate the risk of continuous immunosuppression and AEs, such as the serious infection observed in this trial, by allowing cyclical recovery of immune function. Testing such schedules would require at least 12 months of follow‐up. Sirolimus (rapamycin)'s putative benefits on epigenetic aging, chronic inflammation and body composition likely accrue slowly; short trials risk missing meaningful divergences. Moreover, although our initial data suggests that sirolimus (rapamycin) 6 mg once weekly may blunt early training gains, it may plausibly flatten the longer‐term slope of functional decline. Observing function over a full year would reveal whether longer interdose intervals lead to net preservation of strength and endurance or merely shift the timing of adaptations.

## 5 Conclusion

In sedentary older adults, adding once‐weekly sirolimus (rapamycin) 6 mg to a 13‐week resistance and endurance exercise programme did not enhance functional performance and may instead have attenuated training‐induced gains. The regimen was associated with an increased burden of AEs. Although sirolimus (rapamycin) may still hold geroprotective promise, its use alongside training clearly demands optimisation of timing and confirmation in longer, adequately powered trials. Until such evidence emerges, regular physical activity remains the unequivocal first line strategy for preserving and improving function in older adults.

## Funding

This trial was funded entirely by public donations (crowdfunding) facilitated by Lifespan.io and VitaDAO. The funds were administered by Dr Brad Stanfield Ltd. Neither Lifespan.io nor VitaDAO had any role in the study design, data collection, analysis, decision to publish or preparation of the manuscript.

## Conflicts of Interest

The authors declare no conflicts of interest regarding the study drug or intervention.

B.S. receives advertising revenue from his YouTube channel and is the founder of MicroVitamin, a dietary supplement company; neither entity has commercial ties to sirolimus (rapamycin). He declares no financial affiliation with Pfizer. To ensure independence, data collection was conducted by the Aotearoa Clinical Trials Trust without his influence. M.K. is the Chief Executive Officer of and holds equity in Optispan Inc. J.J. and R.L. are employees of BioValeo, the Contract Research Organization (CRO) commissioned to manage the study. B.L. declares no competing interests. The study drug was purchased commercially from Pfizer; the manufacturer had no input into the trial.

## Supporting information


**Data S1:** Supporting information.
