# Supplementary material for: Exercise and Weekly Sirolimus (Rapamycin) in Older Adults: RAPA‐EX‐01 Randomised, Double‐Blind, Placebo‐Controlled Trial
Source: J Cachexia Sarcopenia Muscle. 2026 Apr 15;17(2):e70274. doi: 10.1002/jcsm.70274 (PMC13082878; doi:10.1002/jcsm.70274)
Supplement: Supplementary file 1 — Data S1: Supporting information. [file JCSM-17-e70274-s001.zip › RAPA-EX-01_Concomitant Medications_V5.0 dated 03Sep2024_.pdf]

Common Medications: Allowed / Prohibited

Medication	NZ Formulary Interaction Information	Allowed / Prohibited
Abametapir	Abametapir might increase the exposure to sirolimus.	Allowed
Adagrasib	Adagrasib is predicted to increase the exposure to sirolimus.	Allowed
Bcg vaccines	Patients who are immunised with live vaccines while receiving immunosuppressants like sirolimus might develop generalised, possibly life-threatening, infections.	prohibited
Belzutifan	Belzutifan is predicted to decrease the exposure to sirolimus.	Allowed
Chikungunya vaccines	Patients who are immunised with live vaccines while receiving immunosuppressants like sirolimus might develop generalised, possibly life-threatening, infections.	prohibited
Dengue vaccines	Patients who are immunised with live vaccines while receiving immunosuppressants like sirolimus might develop generalised, possibly life-threatening, infections.	prohibited
Erdafitinib	Erdafitinib is predicted to affect the exposure to sirolimus.	Allowed
Grapefruit	Grapefruit juice appears to increase the exposure to oral sirolimus.	prohibited
Lasmiditan	Lasmiditan is predicted to increase sirolimus exposure.	Allowed
Live cholera vaccines	Patients who are immunised with live vaccines while receiving immunosuppressants like sirolimus might develop generalised, possibly life-threatening, infections.	prohibited
Live ebola virus vaccines	Patients who are immunised with live vaccines while receiving immunosuppressants like sirolimus might develop generalised, possibly life-threatening, infections.	prohibited

Approver Initials

Live herpes-zoster vaccines	Patients who are immunised with live vaccines while receiving immunosuppressants like sirolimus might develop generalised, possibly life-threatening, infections.	prohibited
Live influenza vaccines	Patients who are immunised with live vaccines while receiving immunosuppressants like sirolimus might develop generalised, possibly life-threatening, infections.	prohibited
Live typhoid vaccines	Patients who are immunised with live vaccines while receiving immunosuppressants like sirolimus might develop generalised, possibly life-threatening, infections.	prohibited
Lumacaftor	Lumacaftor is predicted to decrease sirolimus exposure.	Allowed
Measles, mumps, rubella vaccines	Patients who are immunised with live vaccines while receiving immunosuppressants like sirolimus might develop generalised, possibly life-threatening, infections.	prohibited
Natalizumab	Long-term concurrent use and prior use of immunosuppressants with natalizumab might increase the risk of progressive multifocal leukoencephalopathy (PML).	prohibited
Nirmatrelvir	Nirmatrelvir boosted with ritonavir is predicted to increase the concentration of sirolimus.	Allowed
Nirogacestat	Nirogacestat is predicted to increase the exposure to sirolimus.	Allowed
Pitolisant	Pitolisant is predicted to decrease the exposure to sirolimus.	Allowed
Poliomyelitis vaccines	Patients who are immunised with live vaccines while receiving immunosuppressants like sirolimus might develop generalised, possibly life-threatening, infections.	prohibited
Pralsetinib	Pralsetinib might affect the exposure to sirolimus.	Allowed

Approver Initials

Rotavirus vaccines	Patients who are immunised with live vaccines while receiving immunosuppressants like sirolimus might develop generalised, possibly life-threatening, infections.	prohibited
Rubella vaccines	Patients who are immunised with live vaccines while receiving immunosuppressants like sirolimus might develop generalised, possibly life-threatening, infections.	prohibited
Sparsentan	Sparsentan is predicted to increase the exposure to sirolimus. Sirolimus might increase the risk of angioedema when given with sirolimus.	Allowed
Tepotinib	Tepotinib is predicted to increase the concentration of sirolimus.	Allowed
Varicella-zoster vaccines	Patients who are immunised with live vaccines while receiving immunosuppressants like sirolimus might develop generalised, possibly life-threatening, infections.	prohibited
Yellow fever vaccines	Patients who are immunised with live vaccines while receiving immunosuppressants like sirolimus might develop generalised, possibly life-threatening, infections.	prohibited
Abrocitinib	Abrocitinib might increase the exposure to sirolimus.	Allowed
Amiodarone	A case report describes increased sirolimus concentrations when a paediatric patient was given amiodarone.	Allowed
Apalutamide	Apalutamide is predicted to decrease the exposure to sirolimus.	Allowed
Aprepitant	Sirolimus concentrations appear to be greatly increased by aprepitant.	Allowed
Asciminib	Asciminib is predicted to increase the exposure to sirolimus.	Allowed
Atazanavir	The HIV-protease inhibitors are predicted to increase sirolimus concentrations.	Allowed
Azilsartan	Angioedema has been reported in patients taking sirolimus with losartan. Other angiotensin II receptor antagonists might interact with sirolimus similarly.	Allowed

Approver Initials

Azithromycin	Azithromycin is predicted to increase the exposure to sirolimus.	Allowed
Benazepril	Angioedema has been reported in patients taking sirolimus with ACE inhibitors.	Allowed
Berotralstat	Berotralstat is predicted to increase the concentration of sirolimus.	Allowed
Bezafibrate	The manufacturers say that if sirolimus and bezafibrate are used concurrently the patient should be monitored for rhabdomyolysis.	Allowed
Bosentan	Bosentan is predicted to decrease sirolimus concentrations, and sirolimus might increase bosentan concentrations.	Allowed
Bromocriptine	The UK and US manufacturers predict that bromocriptine will increase sirolimus concentrations.	Prohibited
Bulevirtide	Bulevirtide is predicted to increase the exposure to sirolimus.	Allowed
Candesartan	Angioedema has been reported in patients taking sirolimus with losartan. Other angiotensin II receptor antagonists might interact with sirolimus similarly.	Allowed
Cannabidiol	Cannabidiol is predicted to increase the concentration of sirolimus.	Prohibited
Captopril	Angioedema has been reported in patients taking sirolimus with ACE inhibitors.	Allowed
Carbamazepine	Carbamazepine is predicted to decrease sirolimus concentrations.	Prohibited
Cenobamate	Cenobamate is predicted to decrease the exposure to sirolimus.	Allowed
Ceritinib	Ceritinib is predicted to increase the exposure to sirolimus.	Allowed
And chenodeoxycholic acid	Sirolimus might affect the efficacy of chenodeoxycholic acid.	Allowed
Ciclosporin	Ciclosporin greatly increases sirolimus concentrations, but sirolimus does not appear to alter ciclosporin concentrations. Concurrent long-term use (greater than 3 to 4 months) might increase renal toxicity.	Allowed
Cilazapril	Angioedema has been reported in patients taking sirolimus with ACE inhibitors.	Allowed

Approver Initials

Cimetidine	Cimetidine is predicted to increase sirolimus concentrations.	Prohibited
Ciprofibrate	The manufacturers say that if sirolimus and ciprofibrate are used concurrently the patient should be monitored for rhabdomyolysis.	Allowed
Clarithromycin	A case describes a large increase in sirolimus concentrations and renal impairment on concurrent use.	Prohibited
Clotrimazole	The UK and US manufacturers predict that clotrimazole might increase sirolimus concentrations.	Prohibited (but topical allowed)
Cobicistat	Cobicistat is predicted to increase sirolimus concentrations.	Allowed
Colestilan	Colestilan is predicted to reduce the absorption of sirolimus when given at the same time.	Allowed
Conivaptan	Conivaptan is predicted to increase the exposure to sirolimus.	Allowed
Crizotinib	Crizotinib is predicted to increase the exposure to sirolimus.	Allowed
Daclatasvir	Daclatasvir is predicted to increase the concentration of sirolimus based on it being a substrate for P-glycoprotein, however, the UK manufacturer states that no clinically relevant interaction is predicted to occur.	Allowed
Danazol	The UK and US manufacturers predict that danazol will increase sirolimus concentrations.	Prohibited
Danicopan	Danicopan is predicted to increase the exposure to sirolimus.	Allowed
Daridorexant	Daridorexant is predicted to increase the exposure to sirolimus.	Allowed
Darunavir	The HIV-protease inhibitors are predicted to increase sirolimus concentrations.	Allowed
Delapril	Angioedema has been reported in patients taking sirolimus with ACE inhibitors.	Allowed
Diltiazem	Diltiazem increases sirolimus concentrations. Sirolimus does not appear to affect the pharmacokinetics of diltiazem.	Prohibited

Approver Initials

Doravirine	Doravirine is predicted to decrease sirolimus exposure.	Allowed
Dronedarone	Dronedarone increases sirolimus exposure.	Allowed
Duvelisib	Duvelisib is predicted to increase the exposure to sirolimus.	Allowed
Efavirenz	The UK and US manufacturers of efavirenz predict that it will reduce sirolimus concentrations (by inducing CYP3A4).	Allowed
Elagolix	Elagolix is predicted to affect the exposure to sirolimus.	Allowed
Elbasvir	Elbasvir is predicted to increase the exposure to sirolimus.	Allowed
Eliglustat	Eliglustat is predicted to increase sirolimus exposure.	Allowed
Elranatamab	Elranatamab might affect the exposure to sirolimus.	Allowed
Eluxadoline	Eluxadoline might increase the exposure to sirolimus.	Allowed
Enalapril	Angioedema has been reported in patients taking sirolimus with ACE inhibitors.	Allowed
Enzalutamide	Enzalutamide is predicted to decrease the exposure to sirolimus.	Allowed
Eprosartan	Angioedema has been reported in patients taking sirolimus with losartan. Other angiotensin II receptor antagonists might interact with sirolimus similarly.	Allowed
Erythromycin	Erythromycin moderately increased sirolimus exposure in healthy subjects, and there is a report of two patients who had large elevations in their sirolimus concentrations when they were given erythromycin.	Prohibited
Etravirine	The UK manufacturer of etravirine predicts that it will induce the metabolism of sirolimus (by CYP3A4). However, etravirine is only a weak inducer of CYP3A4.	Allowed
Fedratinib	Fedratinib is predicted to increase the exposure to sirolimus.	Allowed

Approver Initials

Fenofibrate	The manufacturers say that if sirolimus and fenofibrate are used concurrently the patient should be monitored for rhabdomyolysis.	Allowed
Flibanserin	Flibanserin is predicted to increase the exposure to sirolimus.	Allowed
Fosamprenavir	A lower sirolimus dose was needed to maintain therapeutic sirolimus concentrations when it was given with fosamprenavir boosted with ritonavir.	Allowed
Fosinopril	Angioedema has been reported in patients taking sirolimus with ACE inhibitors.	Allowed
Fosnetupitant	Fosnetupitant is predicted to increase the exposure to sirolimus.	Allowed
Fosphenytoin	Several case reports describe increased sirolimus dose requirements in the presence of phenytoin. Fosphenytoin might interact with sirolimus similarly.	Prohibited
Fostamatinib	Fostamatinib is predicted to increase the exposure to sirolimus.	Allowed
Futibatinib	Futibatinib is predicted to increase the exposure to sirolimus.	Allowed
Gemfibrozil	The manufacturers say that if sirolimus and gemfibrozil are used concurrently the patient should be monitored for rhabdomyolysis.	Allowed
Sirolimus givinostat	Givinostat might increase the exposure to sirolimus.	Allowed
Ibrutinib	Ibrutinib might increase the plasma concentration of sirolimus.	Allowed
Idelalisib	Idelalisib is predicted to markedly increase the exposure to sirolimus.	Allowed
Imatinib	Imatinib is predicted to increase the plasma concentration of sirolimus.	Allowed
Imidapril	Angioedema has been reported in patients taking sirolimus with ACE inhibitors.	Allowed
Indinavir	The HIV-protease inhibitors are predicted to increase sirolimus concentrations.	Prohibited
Irbesartan	Angioedema has been reported in patients taking sirolimus with losartan. Other angiotensin II receptor antagonists might interact with sirolimus similarly.	Allowed

Approver Initials

Isavuconazole	Isavuconazole slightly increases sirolimus exposure.	Prohibited (but topical allowed)
Itraconazole	Case reports describe large increases in sirolimus concentrations with itraconazole.	Prohibited (but topical allowed)
Ivacaftor	Ivacaftor is predicted to increase the exposure to sirolimus.	Allowed
Ivosidenib	Ivosidenib might affect the exposure to sirolimus.	Allowed
Ketoconazole	Ketoconazole greatly increases sirolimus concentrations.	Prohibited (but topical allowed)
Lapatinib	Lapatinib is predicted to increase the exposure to sirolimus.	Allowed
Ledipasvir	Ledipasvir is predicted to increase the exposure to sirolimus.	Allowed
Lefamulin	Lefamulin is predicted to increase the exposure to sirolimus.	Allowed
Lesinurad	Lesinurad is predicted to decrease the exposure to sirolimus.	Allowed
Letermovir	Letermovir moderately increases sirolimus exposure.	Prohibited
And levoketoconazole	Levoketoconazole is predicted to increase the exposure to sirolimus.	Prohibited (but topical allowed)
Lisinopril	Angioedema has been reported in patients taking sirolimus with ACE inhibitors.	Allowed
Lomitapide	The manufacturers predict that lomitapide might increase the absorption of sirolimus, by inhibiting P-glycoprotein.	Allowed
Lonafarnib	Lonafarnib is predicted to increase the exposure to sirolimus.	Allowed
Lopinavir	The HIV-protease inhibitors are predicted to increase sirolimus concentrations.	Allowed
Lorlatinib	Lorlatinib might decrease the exposure to sirolimus.	Allowed
Losartan	Angioedema has been reported in patients taking sirolimus with losartan.	Allowed
Maribavir	Maribavir is predicted to increase the exposure to sirolimus.	Allowed
Mavacamten	Mavacamten is predicted to decrease the exposure to sirolimus; the extent of the interaction varies depending on an individual's CYP2C19 metaboliser status.	Allowed

Approver Initials

Mavorixafor	Mavorixafor is predicted to increase the exposure to sirolimus.	Allowed
Miconazole	Miconazole oral gel might be absorbed in sufficient quantities to increase sirolimus concentrations.	Allowed
Mifepristone	Mifepristone is predicted to increase the exposure to sirolimus.	Allowed
Mitapivat	Mitapivat is predicted to decrease the exposure to sirolimus.	Allowed
Mitotane	Mitotane is predicted to decrease the exposure to sirolimus.	Allowed
Mobocertinib	Mobocertinib is predicted to decrease the exposure to sirolimus.	Allowed
Moexipril	Angioedema has been reported in patients taking sirolimus with ACE inhibitors.	Allowed
Mycophenolate	Higher concentrations of mycophenolic acid have been seen in kidney transplant patients taking mycophenolate with sirolimus, when compared with similar patients taking mycophenolate with ciclosporin.	Allowed
Neratinib	Neratinib is predicted to increase the exposure to sirolimus.	Allowed
Netupitant	Netupitant is predicted to increase sirolimus exposure.	Allowed
Nevirapine	The US manufacturer of nevirapine predicts that it will reduce sirolimus concentrations (by inducing CYP3A4).	Allowed
Nilotinib	Nilotinib is predicted to increase sirolimus exposure.	Allowed
Olaparib	Olaparib might increase the exposure to sirolimus.	Allowed
Olmesartan	Angioedema has been reported in patients taking sirolimus with losartan. Other angiotensin II receptor antagonists might interact with sirolimus similarly.	Allowed
Olutasidenib	Olutasidenib is predicted to decrease the exposure to sirolimus.	Allowed
Pacritinib	Pacritinib might affect the exposure to sirolimus.	Allowed

Approver Initials

Paritaprevir	The exposure to sirolimus appears to be very markedly increased by paritaprevir boosted with ritonavir (in a fixed-dose combination with ombitasvir, and given with dasabuvir).	prohibited
Pemigatinib	Pemigatinib might increase the exposure to sirolimus.	Allowed
Perindopril	Angioedema has been reported in patients taking sirolimus with ACE inhibitors.	Allowed
Pexidartinib	Pexidartinib is predicted to decrease the concentration of sirolimus.	Allowed
Phenobarbital	Phenobarbital is predicted to decrease sirolimus concentrations.	Allowed
Phenytoin	Several case reports describe increased sirolimus dose requirements in the presence of phenytoin.	Prohibited
Pibrentasvir	Pibrentasvir is predicted to increase the exposure to sirolimus.	Allowed
Pirtobrutinib	Pirtobrutinib is predicted to increase the exposure to sirolimus.	Allowed
Posaconazole	Posaconazole greatly increases sirolimus concentrations.	Prohibited (but topical allowed)
Primidone	Phenobarbital is predicted to decrease sirolimus concentrations. Primidone is metabolised to phenobarbital and is expected to interact similarly.	Allowed
Quinapril	Angioedema has been reported in patients taking sirolimus with ACE inhibitors.	Allowed
Quinidine	Quinidine is predicted to increase the exposure to sirolimus.	Allowed
Ramipril	Angioedema has been reported in patients taking sirolimus with ACE inhibitors.	Allowed
Ranolazine	The manufacturers predict that ranolazine will increase sirolimus levels.	Allowed
Repotrectinib	Repotrectinib is predicted to decrease the exposure to sirolimus.	Allowed
Ribociclib	Ribociclib is predicted to increase sirolimus exposure.	Allowed
Rifabutin	Rifabutin is predicted to decrease sirolimus concentrations.	Prohibited
Rifampicin	Rifampicin greatly decreases sirolimus concentrations.	Prohibited

Approver Initials

Rifapentine	Rifapentine is predicted to decrease sirolimus concentrations.	Allowed
Ritonavir	A lower sirolimus dose was needed to maintain therapeutic sirolimus concentrations when it was given with low-dose ritonavir (with fosamprenavir).	Allowed
Rolapitant	Rolapitant is predicted to increase the exposure to sirolimus.	Allowed
Rufinamide	Rufinamide is predicted to decrease the exposure to sirolimus.	Allowed
Saquinavir	The HIV-protease inhibitors are predicted to increase sirolimus concentrations.	Allowed
Sarecycline	Sarecycline is predicted to increase the exposure to sirolimus.	Allowed
Sarilumab	Sarilumab is predicted to affect the exposure to sirolimus.	Allowed
Satralizumab	Satralizumab might increase the exposure to sirolimus.	Allowed
Selpercatinib	Selpercatinib is predicted to increase the exposure to sirolimus.	Allowed
Sorafenib	Clinically important toxicities were seen in a phase I study of sirolimus and sorafenib.	probihited
Sotorasib	Sotorasib is predicted to decrease the exposure to sirolimus.	Allowed
Spirapril	Angioedema has been reported in patients taking sirolimus with ACE inhibitors.	Allowed
St john's wort	St John's wort is predicted to decrease sirolimus concentrations.	Prohibited
Stiripentol	The UK manufacturer predicts that stiripentol might inhibit the metabolism of sirolimus.	Allowed
Sunitinib	A pharmacokinetic study found that sunitinib exposure is increased by sirolimus; the pharmacokinetics of sirolimus remain unaltered.	Allowed
Tacrolimus	The concurrent use of sirolimus and tacrolimus might reduce tacrolimus concentrations and might increase sirolimus exposure. Increased adverse effects might occur.	Allowed
Telithromycin	Telithromycin is predicted to greatly increased sirolimus concentrations.	Allowed

Approver Initials

Telmisartan	Angioedema has been reported in patients taking sirolimus with losartan. Other angiotensin II receptor antagonists might interact with sirolimus similarly.	Allowed
Tipranavir	The HIV-protease inhibitors are predicted to increase sirolimus concentrations.	Allowed
Tofisopam	Tofisopam is predicted to increase the exposure to sirolimus.	Allowed
Tolvaptan	Tolvaptan is predicted to increase the exposure to sirolimus.	Allowed
Tovorafenib	Tovorafenib is predicted to decrease the exposure to sirolimus.	Allowed
Trandolapril	Angioedema has been reported in patients taking sirolimus with ACE inhibitors.	Allowed
Treosulfan	Treosulfan might affect the exposure to sirolimus.	Allowed
Trofinetide	Trofinetide might increase the exposure to sirolimus.	Allowed
Tucatinib	Tucatinib is predicted to increase the exposure to sirolimus.	Allowed
Ulipristal	Ulipristal is predicted to increase sirolimus concentrations.	Allowed
Valbenazine	Valbenazine is predicted to increase the exposure to sirolimus.	Allowed
Valsartan	Angioedema has been reported in patients taking sirolimus with losartan. Other angiotensin II receptor antagonists might interact with sirolimus similarly.	Allowed
Valspodar	Valspodar is predicted to increase the exposure to sirolimus.	Allowed
Vandetanib	Vandetanib is predicted to increase sirolimus exposure.	Allowed
Velpatasvir	Velpatasvir is predicted to increase the exposure to sirolimus.	Allowed
Vemurafenib	The manufacturers predict that vemurafenib might increase the exposure to sirolimus (by inhibiting P-glycoprotein).	Allowed
Venetoclax	Venetoclax is predicted to increase the exposure to sirolimus.	Allowed

Approver Initials

Verapamil	Verapamil increases sirolimus concentrations. Sirolimus causes a small increase in verapamil concentrations.	Probihited
Voclosporin	Voclosporin is predicted to increase the exposure to sirolimus.	Allowed
Voriconazole	Voriconazole greatly increases sirolimus concentrations.	Prohibited (but topical allowed)
Voxelotor	Voxelotor is predicted to increase the exposure to sirolimus.	Allowed
Zanubrutinib	Zanubrutinib is predicted to affect the exposure to sirolimus.	Allowed
Zofenopril	Angioedema has been reported in patients taking sirolimus with ACE inhibitors.	Allowed
Atorvastatin	The concurrent use of atorvastatin and sirolimus does not generally alter the pharmacokinetics of either drug. Some evidence suggests a higher incidence of myopathy in transplant patients taking statins and sirolimus. Cases of pneumonitis have been reported.	Allowed
Avacopan	Avacopan is predicted to increase the the exposure to sirolimus.	Allowed
Brigatinib	Brigatinib is predicted to affect the exposure to sirolimus.	Allowed
Cabozantinib	Cabozantinib might increase the exposure to sirolimus.	Allowed
Cariprazine	Cariprazine is predicted to increase the exposure to sirolimus, but the clinical relevance is unknown.	Allowed
Dabrafenib	Dabrafenib is predicted to affect the exposure to sirolimus.	Allowed
Dasatinib	Dasatinib is predicted to increase the exposure to sirolimus.	Allowed
And dexlansoprazole	Proton pump inhibitors can cause hypomagnesaemia, which might be additive with the magnesium-lowering effects of sirolimus.	Allowed
Enasidenib	Enasidenib is predicted to increase the exposure to sirolimus.	Allowed
Esomeprazole	Proton pump inhibitors can cause hypomagnesaemia, which might be additive with the magnesium-lowering effects of sirolimus.	Allowed

Approver Initials

Fluconazole	A study and two case reports, one of which was fatal, describe large increases in sirolimus concentrations with fluconazole, despite dose reductions.	Prohibited (but topical allowed)
Fluvastatin	Fluvastatin has been reported to cause rhabdomyolysis when given with sirolimus.	prohibited
Gilteritinib	Gilteritinib is predicted to increase the exposure to sirolimus.	Allowed
Glasdegib	Glasdegib is predicted to increase the exposure to sirolimus.	Allowed
Glycerol phenylbutyrate	Glycerol phenylbutyrate might decrease the exposure to sirolimus.	Allowed
Lansoprazole	Proton pump inhibitors can cause hypomagnesaemia, which might be additive with the magnesium-lowering effects of sirolimus.	Allowed
Larotrectinib	Larotrectinib is predicted to increase the exposure to sirolimus.	Allowed
Lenacapavir	Lenacapavir is predicted to increase the concentration of sirolimus.	Allowed
Lovastatin	Some evidence suggests a higher incidence of myopathy in transplant patients taking statins and sirolimus.	Allowed
And metoclopramide	The UK and US manufacturers predict that metoclopramide might increase sirolimus concentrations.	Prohibited
Micafungin	Sirolimus does not affect the pharmacokinetics of micafungin. Micafungin might increase the exposure to sirolimus.	Allowed
Mirabegron	Mirabegron is predicted to slightly increase sirolimus exposure.	Allowed
Momelotinib	Momelotinib might increase the exposure to sirolimus.	Allowed
Nicardipine	The UK and US manufacturers predict that nicardipine might increase sirolimus concentrations.	Allowed
Omeprazole	Proton pump inhibitors can cause hypomagnesaemia, which might be additive with the magnesium-lowering effects of sirolimus.	Allowed
Palbociclib	Palbociclib is predicted to increase the exposure to sirolimus.	Allowed

Approver Initials

Pantoprazole	Proton pump inhibitors can cause hypomagnesaemia, which might be additive with the magnesium-lowering effects of sirolimus.	Allowed
Piperaquine	Piperaquine is predicted to increase the exposure to sirolimus.	Allowed
Pitavastatin	Some evidence suggests a higher incidence of myopathy in transplant patients taking statins and sirolimus.	Allowed
Ponatinib	Ponatinib is predicted to increase the concentration of sirolimus.	Allowed
Pravastatin	Some evidence suggests a higher incidence of myopathy in transplant patients taking statins and sirolimus.	Allowed
Rabeprazole	Proton pump inhibitors can cause hypomagnesaemia, which might be additive with the magnesium-lowering effects of sirolimus.	Allowed
Remdesivir	Remdesivir might transiently increase the concentration of sirolimus.	Allowed
Ripretinib	Ripretinib is predicted to increase the exposure to sirolimus (UK, EU).	Allowed
Ritlecitinib	Ritlecitinib is predicted to increase the exposure to sirolimus.	Allowed
Rosuvastatin	Some evidence suggests a higher incidence of myopathy in transplant patients taking statins and sirolimus.	Allowed
Rucaparib	Rucaparib is predicted to increase the exposure to sirolimus. The clinical relevance is unknown.	Allowed
Simvastatin	Simvastatin has been reported to cause rhabdomyolysis when given with sirolimus.	Prohibited
Tocilizumab	Tocilizumab is predicted to increase the metabolism of drugs metabolised by CYP3A4, such as sirolimus.	Allowed
Ublituximab	Sirolimus might increase the risk of serious infection when given with ublituximab.	Allowed
And ursodoxicoltaurine	Ursodoxicoltaurine with sodium phenylbutyrate is predicted to affect the exposure to sirolimus.	Allowed
Vonoprazan	Vonoprazan is predicted to increase the exposure to sirolimus.	Allowed

Approver Initials

Aciclovir	No pharmacokinetic interaction occurs between aciclovir and sirolimus.	Allowed
Betamethasone	Corticosteroids do not appear to have a clinically relevant effect on sirolimus minimum concentrations.	Allowed
Carfilzomib	Based on in vitro data, carfilzomib might increase the exposure to sirolimus.	Allowed
Combined hormonal contraceptives	Sirolimus does not appear to affect the pharmacokinetics of combined hormonal contraceptives containing ethinylestradiol and norgestrel. Sirolimus might be teratogenic.	Allowed
Cortisone	Corticosteroids do not appear to have a clinically relevant effect on sirolimus minimum concentrations.	Allowed
Deflazacort	Corticosteroids do not appear to have a clinically relevant effect on sirolimus minimum concentrations.	Allowed
And dexamethasone	Corticosteroids do not appear to have a clinically relevant effect on sirolimus minimum concentrations.	Allowed
Digoxin	No pharmacokinetic interaction occurs between digoxin and sirolimus.	Allowed
Elacestrant	Elacestrant is predicted to increase the exposure to sirolimus.	Allowed
Encorafenib	Encorafenib is predicted to affect the concentration of sirolimus.	Allowed
Entrectinib	Entrectinib is predicted to increase the exposure to sirolimus.	Allowed
Eribulin	Eribulin might increase the concentration of sirolimus (UK). However, a clinically relevant interaction seems unlikely.	Allowed
Everolimus	Everolimus is predicted to increase sirolimus concentrations, but this is not expected to be clinically relevant.	Allowed
Ezetimibe	The concurrent use of ezetimibe and sirolimus does not result in any clinically relevant pharmacokinetic changes.	Allowed
Sirolimus and food	A high-fat meal slightly increased the exposure to sirolimus in one study.	Allowed
Glibenclamide	No pharmacokinetic interaction occurs between glibenclamide and sirolimus.	Allowed

Approver Initials

Hydrocortisone	Corticosteroids do not appear to have a clinically relevant effect on sirolimus minimum concentrations.	Allowed
Idebenone	Idebenone is predicted to increase the exposure to sirolimus.	Allowed
Istradefylline	Istradefylline is predicted to increase the exposure to sirolimus.	Allowed
And methylprednisolone	Intravenous methylprednisolone appears to have no effect on sirolimus minimum concentrations.	Allowed
Nifedipine	No pharmacokinetic interaction appears to occur between nifedipine and sirolimus.	Allowed
Niraparib	Niraparib might affect sirolimus exposure.	Allowed
Omega-3 fatty acids	Omega-3 fatty acids might increase the concentration of sirolimus.	Allowed
Prednisolone	Corticosteroids do not appear to have a clinically relevant effect on sirolimus minimum concentrations.	Allowed
Prednisone	Sirolimus minimally increases prednisolone concentrations (derived from prednisone), which is not expected to be clinically relevant.	Allowed
Pretomanid	Pretomanid is predicted to increase the exposure to sirolimus.	Allowed
Repaglinide	Repaglinide does not appear to alter sirolimus concentrations.	Allowed
Ruxolitinib	The UK manufacturer predicts that ruxolitinib might increase the exposure to P-glycoprotein substrates, such as sirolimus, but the US manufacturer states that ruxolitinib does not inhibit P-glycoprotein.	Allowed
And sulfamethoxazole	A single dose of co-trimoxazole (which contains sulfamethoxazole) did not alter sirolimus concentrations in one study.	Allowed
Tazemetostat	Tazemetostat is predicted to decrease the exposure to sirolimus.	Allowed
Triamcinolone	Corticosteroids do not appear to have a clinically relevant effect on sirolimus minimum concentrations.	Allowed
Trimethoprim	A single dose of co-trimoxazole (which contains trimethoprim) did not alter sirolimus concentrations in one study.	Allowed

Approver Initials

Zonisamide	Zonisamide is a weak inhibitor of P-glycoprotein. The manufacturers therefore predict that it might affect the pharmacokinetics of other P-glycoprotein substrates, such as sirolimus.	Allowed

**Medical Monitor Approval:**	Name, signature	**Date:**	

Approver Initials
