# Supplementary material for: Exercise and Weekly Sirolimus (Rapamycin) in Older Adults: RAPA‐EX‐01 Randomised, Double‐Blind, Placebo‐Controlled Trial
Source: J Cachexia Sarcopenia Muscle. 2026 Apr 15;17(2):e70274. doi: 10.1002/jcsm.70274 (PMC13082878; doi:10.1002/jcsm.70274)
Supplement: Supplementary file 1 — Data S1: Supporting information. [file JCSM-17-e70274-s001.zip › Ethics Approval.pdf]

Health and Disability Ethics Committees
Ministry of Health
133 Molesworth Street
PO Box 5013
Wellington
6011
hdecs@health.govt.nz

Ethics reference: 2024 FULL 20084

17 June 2024

Dr Joanna Wojciechowska

100 Hospital Rd
Auckland
2025
New Zealand

Tēnā koe Dr Wojciechowska

APPROVAL OF APPLICATION

Study title: A single-centre, double-blind, randomized, placebo-controlled, 2-arm study to evaluate safety and efficacy of intermittent Rapamycin on muscle strength and endurance in older adults following a 13-week exercise program

I am pleased to advise that your application was **approved** by the Northern B Health and Disability Ethics Committee (the Committee) with non-standard conditions. This decision was made through the FULL pathway.

Conditions of HDEC approval

HDEC approval for this study is subject to the following conditions being met prior to the commencement of the study in New Zealand. It is your responsibility, and that of the study's sponsor, to ensure that these conditions are met. No further review by the Northern B Health and Disability Ethics Committee is required.

Standard conditions:

- Before the study commences at *any* locality in New Zealand, all relevant regulatory approvals must be obtained.
- Before the study commences at *any* locality in New Zealand, it must be registered in a clinical trials registry. This should be a registry approved by the World Health Organization (such as the Australia New Zealand Clinical Trials Registry, www.anzctr.org.au or https://clinicaltrials.gov/).
- Before the study commences at *each given* locality in New Zealand, it must be authorised by that locality in Ethics RM. Locality authorisation confirms that the locality is suitable for the safe and effective conduct of the study, and that local research governance issues have been addressed.

Non-standard conditions:

- The provisional approval letter requested that 'all adverse events should be reported, with an assessment made by the Investigator or designee as to the severity and relatedness of the event. Please amend the protocol to ensure collection of adverse event data is in line with standard practice'.
  The request has not been actioned. It is still proposed not to collect AEs except those that meet the definition of an SAE, impact exercise ability or dosing. Rapamycin is currently approved for the treatment of a serious and potentially life-threatening condition i.e., the prevention of rejection of renal transplants.
  The potential benefits to be assessed in the proposed trial are of significantly more minor nature. Data on minor adverse events should therefore also be collected to allow the value of minor benefits, such as an increase in the effectiveness of exercise, to be assessed against the frequency of non-serious AEs.

Non-standard conditions must be completed before commencing your study, however, they do not need to be submitted to or reviewed by HDECs.

If you would like an acknowledgement of completion of your non-standard conditions you may submit a post approval form amendment through the Ethics Review Manager. Please clearly identify in the amendment form that the changes relate to non-standard conditions and ensure that supporting documents (if requested) are tracked/highlighted with changes.

For information on non-standard conditions please see paragraphs 125 and 126 of the *Standard Operating Procedures for Health and Disability Ethics Committees (SOPs)*.

After HDEC review

Please refer to the *SOPs* for HDEC requirements relating to amendments and other post-approval processes.

Your next progress report is due by 11 June 2025.

For the avoidance of doubt, Development Safety Update Reports may serve as annual safety reports to HDECs provided that they contain the information outlined above. These summaries should be accompanied by comment from the New Zealand coordinating investigator of the study.

Please refer to paragraphs 206 to 208 of the *SOPs* for further information.

Participant access to compensation

The Northern B Health and Disability Ethics Committee is satisfied that your study is not a clinical trial that is to be conducted principally for the benefit of the manufacturer or distributor of the medicine or item being trialed. Participants injured as a result of treatment received as part of your study may therefore be eligible for publicly-funded compensation through the Accident Compensation Corporation.

Further information and assistance

Please contact the HDECs Secretariat at hdecs@health.govt.nz or visit our website at www.ethics.health.govt.nz for more information, as well as our General FAQ and Ethics RM user manual.

Nāku noa, nā

Ms Kate O'Connor

Chair

Northern B Health and Disability Ethics Committee

Encl: Appendix A: documents submitted

Appendix B: statement of compliance and list of members

Appendix A: Documents submitted

Document Type	File Name	Date	Version
Scientific Peer Review	RAPA-EX-01_hdec-peer-review-kaeberlein, v1.0	05/03/2022	1.0
Response to PA Document	RAPA-EX-01_hdec-peer-review-kaeberlein, v1.0	05/03/2022	1.0
CV for Coordinating Investigator	ACTT_Wojciechowska J_CV SIGNED_2023_AUG_31	31/08/2023	
Non-Review Document	rapamune CMI summary - Medsafe	01/12/2023	Medsafe
Surveys/questionnaires	RAPA-EX-01_ SF36 questionnaire_ v1.0_ 31Mar2024	31/03/2024	1.0
Advertisement	RAPA-EX-01, Rapamycin Script, v1.0, 01Apr2024	01/04/2024	1.0
Advertisement	RAPA-EX-01_Pre-screen questionnaire V1.0_ 01Apr2024	01/04/2024	1.0
Protocol	RAPA-EX-01_Protocol_V1.0_02Apr2024	02/04/2024	1.0
Other	RAPA-EX-01_Participant Daily Diary_PART 1_V1.0_03Apr2024	03/04/2024	1
Other	RAPA-EX-01_Participant Daily Diary_PART 2_V1.0_03Apr2024	03/04/2024	1
Other	RAPA-EX-01_Participant Daily Diary_PART 2_V1.0_03Apr2024	03/04/2024	1.0
Other	RAPA-EX-01_Participant Daily Diary_PART 1_V1.0_03Apr2024	03/04/2024	1.0
Other	RAPA-EX-01_Participant Daily Diary_PART 1_V1.0_03Apr2024	03/04/2024	1.0
PIS/CF	RAPA-EX-01_PICF_V1.0 _ 4Apr2024	04/04/2024	1.0
Data and Tissue Management Plan	RAPA-EX-01_HDEC-data-tissue-management_ v1.0_04Apr24	04/04/2024	1.0
Non-Review Document	RAPA-EX-01_Participant Card_V1.0 _ 10Apr2024	10/04/2024	1.0
Response to PA Document	RAPA-EX-01_PICF_V1.0 _ 21May2024	21/05/2024	1.0
Response to PA Document	RAPA-EX-01_PICF_V1.0 _ 21May2024_TC	21/05/2024	1.0
Response to PA Document	RAPA-EX-01_MK_hdec-peer-review_24May2024	24/05/2024	1.0
Response to PA Document	RAPA-EX-01_Protocol_V1.0_01June2024	01/06/2024	1.0
Response to PA Document	RAPA-EX-01_Protocol_V1.0_01June2024_TC	01/06/2024	1.0
Response to PA Document	RAPA-EX-01_ B.Arroll_hdec-peer-review_2Jun2024	02/06/2024	1.0
Response to PA Document	RAPA-EX-01_ Provisional approval response_05June2024	05/06/2024	1.0
Response to PA Document	RAPA-Ex-01_ACTT Advertising Template_v1.0_5June24	05/06/2024	1.0
Response to PA Document	RAPA-EX-01, Rapamycin Script, v1.0, 05June2024	05/06/2024	1.0
Response to PA Document	RAPA-EX-01, Rapamycin Script, v1.0, 05June2024_TC	05/06/2024	1.0

Appendix B: Statement of compliance and list of members

Statement of compliance

The Northern B Health and Disability Ethics Committee

- is constituted in accordance with its Terms of Reference
- operates in accordance with the *Standard Operating Procedures for Health and Disability Ethics Committees*, and with the principles of international good clinical practice (GCP)
- is approved by the Health Research Council of New Zealand's Ethics Committee for the purposes of section 25(1)(c) of the Health Research Council Act 1990
- is registered (number 00008715) with the US Department of Health and Human Services' Office for Human Research Protection (OHRP).

List of members

Ms Kate O'Connor (Lay (ethical/moral reasoning)), Mrs Leesa Russell (Non-lay (observational/intervention studies)), Mr Barry Taylor (Non-lay (observational/intervention studies)), Ms Alice McCarthy (Lay (the law)), Ms Joan Pettit (Non-lay (intervention studies)), Ewe Leong Lim (Lay (consumer/community perspectives)), Maakere Marr (Lay (consumer/community perspectives)).

Unless members resign, vacate or are removed from their office, every member of HDEC shall continue in office until their successor comes into office (HDEC Terms of Reference).

http://www.ethics.health.govt.nz
