# Supplementary material for: Exercise and Weekly Sirolimus (Rapamycin) in Older Adults: RAPA‐EX‐01 Randomised, Double‐Blind, Placebo‐Controlled Trial
Source: J Cachexia Sarcopenia Muscle. 2026 Apr 15;17(2):e70274. doi: 10.1002/jcsm.70274 (PMC13082878; doi:10.1002/jcsm.70274)
Supplement: Supplementary file 1 — Data S1: Supporting information. [file JCSM-17-e70274-s001.zip › RAPA-EX-01_Participant Daily Diary_PART 2_V1.1_15Aug2024-DI.pdf]

Daily Diary

Part 2

A Single-Centre, Double-Blind, Randomized, Placebo-Controlled 2-Arm Study to Evaluate Safety and Efficacy of Intermittent Rapamycin On Muscle Strength and Endurance In Older Adults Following A 13-Week Exercise Program (RAPA-EX-01)

**Participant details**
**Participant Study Number**:
Participant Sticker

Key Contact Details

Study Site: Study Coordinator - <<Name>>

Email: <<email>>

Tel: <<phone>>

Rutherford Fitness:

Tel: <<phone>>

Email: <<email>>

Participant Daily Diary

Thank you for participating in this investigation on the impact of Rapamycin on Muscle Strength and Endurance

Please complete this diary each day, recording the exercise you perform, your weekly study treatment dosing, and any variations from your exercise or dosing programme.

During this study, you are asked to complete a 13-week, thrice-weekly (3x per week) exercise program and to take a weekly dose of study treatment.

The exercise program changes each week. The exercise program has 2 parts.

You will always start your exercise program with the chair stand, followed by the exercycle.

2-part exercise program overview:

Part 1 **30-Second Chair Stand**

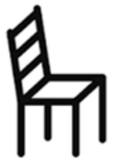

The chair stand is the same every time.

Perform the chair stand by sitting and standing from the chair as many times as possible for 30 seconds.

Part 2 **Exercycle**

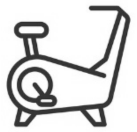

The exercycle program changes to increase the intensity and duration of the workout until the required level is achieved.

Your exercycle program will consist of a 2-minute warm-up, training, and a 2-minute cool down.

30-Second Chair Stand

The chair stand should be performed using a stable chair without arms, such as a dining room chair.

The chair can be placed against a wall to prevent it from moving.

1. Sit in the middle of the chair, back straight with feet approximately shoulder width apart.
2. Arms are crossed at the wrists and held against the chest.
3. Complete as many full stands as possible within 30 seconds. Sit fully between each stand.
4. Count the number of full chair stands performed.

The below link is for a video that provides guidance on correct technique and performance on the 30 second chair stand test.

https://www.physio-pedia.com/30_Seconds_Sit_To_Stand_Test

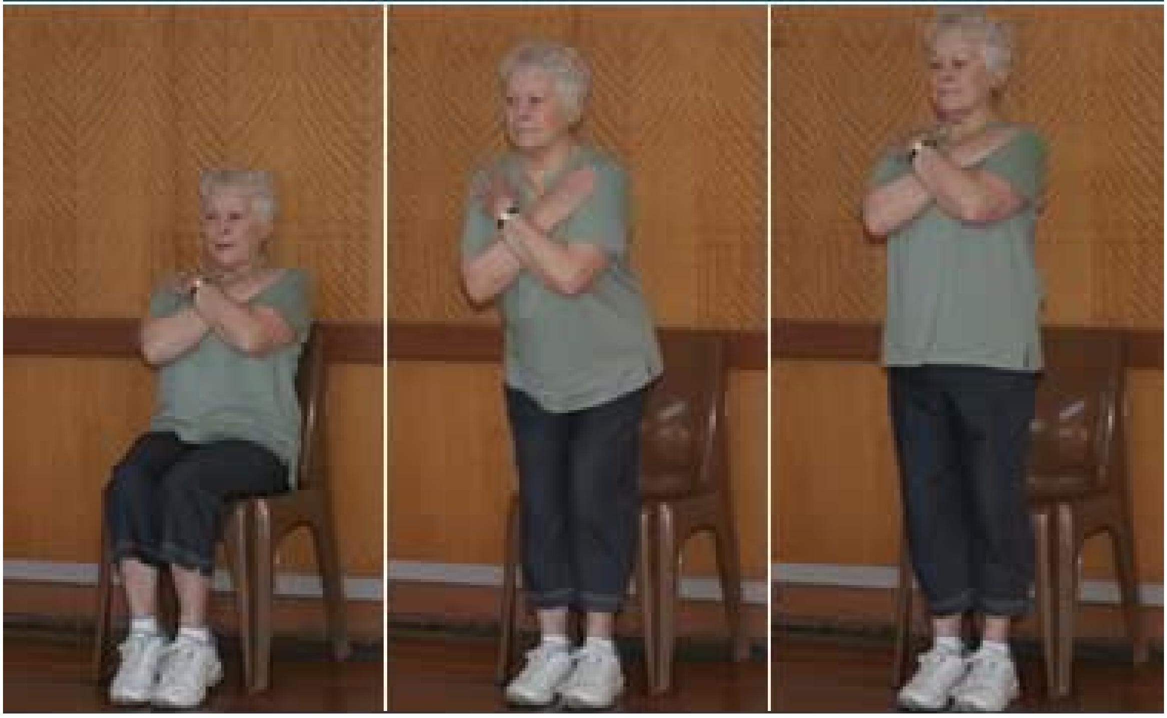

Exercycle

You will be provided with an exercycle to use for the duration of the study.

The exercycle is supplied by Rutherford Fitness.

Rutherford Fitness will deliver the exercycle and explain how to use it.

The exercycle program changes over the course of the study, until the required level is achieved.

Below is an overview of study exercise programme.

Week	Warm-up	Training	Cooldown	Total exercycle time
1	2 min,<br>at 17 km/h,<br>on resistance level 1	10 min,<br>at 25-28 km/h,<br>on resistance level 1	2 min,<br>at 16 km/h,<br>on resistance level 1	14 min
2	2 min,<br>at 17 km/h,<br>on resistance level 1	15 min,<br>at 25-28 km/h,<br>on resistance level 1	2 min,<br>at 16 km/h,<br>on resistance level 1	19 min
3	2 min,<br>at 17 km/h,<br>on resistance level 1	20 min,<br>at 25-28 km/h,<br>on resistance level 2	2 min,<br>at 16 km/h,<br>on resistance level 1	24 min
4	2 min,<br>at 17 km/h,<br>on resistance level 1	25 min,<br>at 25-28 km/h,<br>on resistance level 2	2 min,<br>at 16 km/h,<br>on resistance level 1	29 min
5	2 min,<br>at 17 km/h,<br>on resistance level 1	25 min,<br>at 25-28 km/h,<br>on resistance level 3	2 min,<br>at 16 km/h,<br>on resistance level 1	29 min
6	2 min,<br>at 17 km/h,<br>on resistance level 1	25 min,<br>at 25-28 km/h,<br>on resistance level 3	2 min,<br>at 16 km/h,<br>on resistance level 1	29 min
7	2 min,<br>at 17 km/h,<br>on resistance level 1	25 min,<br>at 25-28 km/h,<br>on resistance level 4	2 min,<br>at 16 km/h,<br>on resistance level 1	29 min
8	2 min,<br>at 17 km/h,<br>on resistance level 1	25 min,<br>at 25-28 km/h,<br>on resistance level 4	2 min,<br>at 16 km/h,<br>on resistance level 1	29 min
9	2 min,<br>at 17 km/h,<br>on resistance level 1	25 min,<br>at 25-28 km/h,<br>on resistance level 5	2 min,<br>at 16 km/h,<br>on resistance level 1	29 min
10	2 min,<br>at 17 km/h,<br>on resistance level 1	25 min,<br>at 25-28 km/h,<br>on resistance level 5	2 min,<br>at 16 km/h,<br>on resistance level 1	29 min
11	2 min,<br>at 17 km/h,<br>on resistance level 1	25 min,<br>at 25-28 km/h,<br>on resistance level 5	2 min,<br>at 16 km/h,<br>on resistance level 1	29 min
12	2 min,<br>at 17 km/h,<br>on resistance level 1	25 min,<br>at 25-28 km/h,<br>on resistance level 5	2 min,<br>at 16 km/h,<br>on resistance level 1	29 min
13	2 min,<br>at 17 km/h,<br>on resistance level 1	25 min,<br>at 25-28 km/h,<br>on resistance level 5	2 min,<br>at 16 km/h,<br>on resistance level 1	29 min

The time and km/h will be displayed on the exercycle screen.

To achieve the correct speed (km/h), you will need to adjust the speed at which you are pedalling.

Occasionally going above or below the km/h is normal and does not need to be recorded, but you should aim to remain within the assigned km/h limit for the majority of the assigned time.

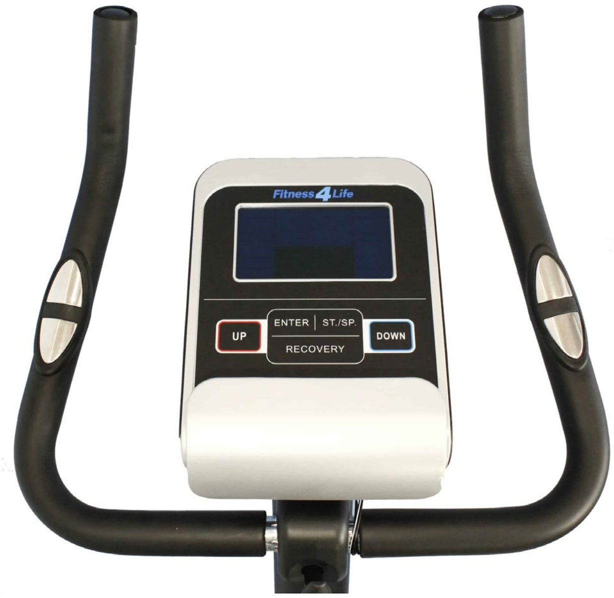

The km/h that you achieve will be displayed on the screen.

The time that you pedal for will also be displayed on the screen.

Each session will start with a 2 min warm up period, followed by the training period at the speed and resistance assigned by your diary for that day, followed by a 2 min cool-down period.

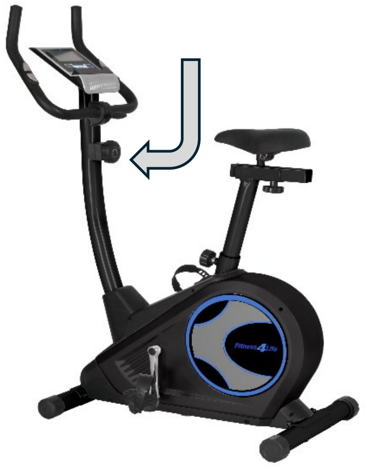

The resistance can be altered by selecting the correct number on the dial under the handlebars (indicated by arrow).

Study Treatment

You will not know whether you have been assigned to receive Rapamycin or placebo.

The Rapamycin or placebo capsules will appear the same.

- The treatment will be contained within a blister pack.
- Each dose will consist of 3 capsules.
- Study treatment is taken once per week.
- You will take your study treatment on day 6 of your weekly schedule, in the morning at breakfast time.
- Record your study dosing within your daily diary.

- *If you miss a dose or take too many or too few capsules, please contact your study site.*

Your study treatment should be stored below 25°C, protected from light and moisture.

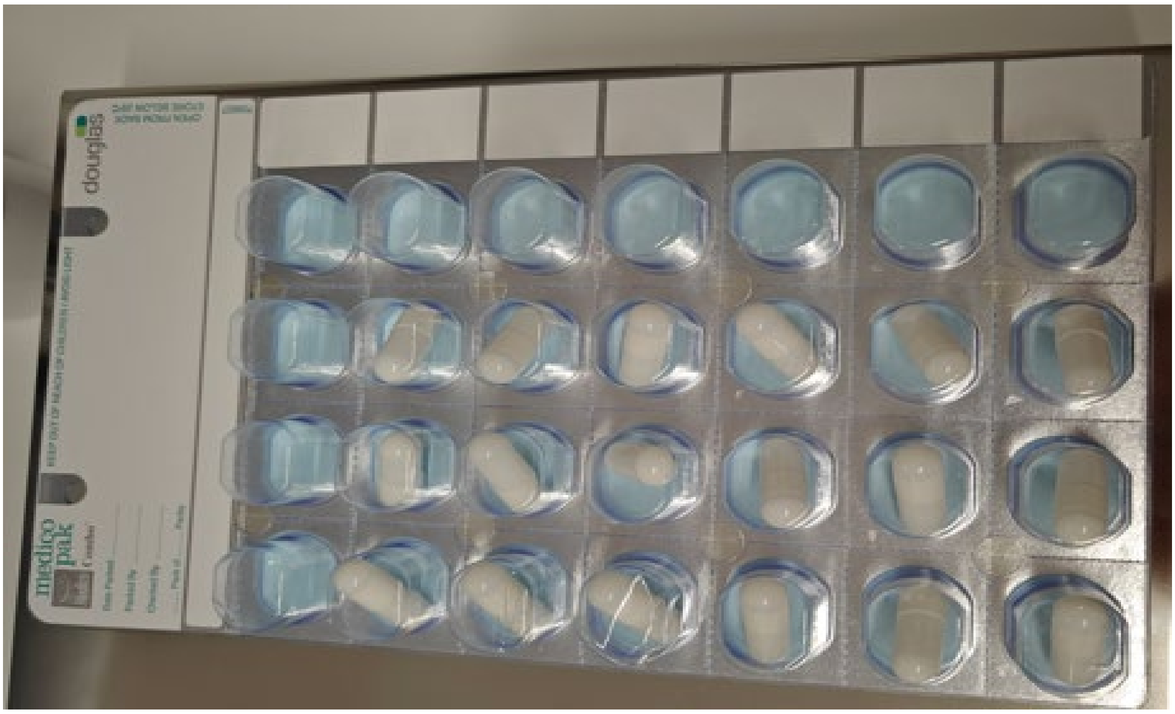

Week 7, Day 1 Weekday & Date: ________________

Today's Exercise Schedule

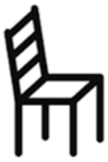

Part 1: Chair stands

Perform the chair stand by sitting and standing from a chair as many times as possible for 30 seconds.

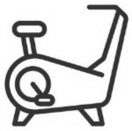

Part 2: Exercycle

Warm-up: 2 min, at 17 km/h, on resistance level 1
Training: 25 min, at 25 -28 km/h, on resistance level 4
Cool down: 2 min, at 16 km/h, on resistance level 1

**Scheduled Exercise**	(Circle answer)
Did you complete both parts of today's exercise program as indicated?	Yes / No
**If NO:**	(Circle answer)
Did you partially complete the program?	Yes / No
Please describe the exercise you managed, on the exercycle:<br>• Length of exercise time:<br>• Training km/h:<br>• Resistance setting:	Time:<br><br>km/h:<br><br>Resistance:
Did you not complete your set exercise program because you were injured or unwell?	Yes / No
If so, please briefly describe any illness or injuries that impacted your ability to exercise:	
**Un-Scheduled Exercise**<br>Exercise performed in addition to the provided study program	
Please briefly list the type of additional exercise performed and time spent.	

Week 7, Day 2 Weekday & Date: ________________

Rest Day

Un-Scheduled Exercise<br>Exercise performed in addition to the provided study program
Please briefly list the type of additional exercise performed and time spent.

Week 7, Day 3 Weekday & Date: ________________

Today's Exercise Schedule

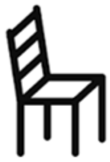

Part 1: Chair stands

Perform the chair stand by sitting and standing from a chair as many times as possible for 30 seconds.

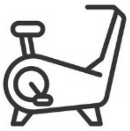

Part 2: Exercycle

Warm-up: 2 min, at 17 km/h, on resistance level 1
Training: 25 min, at 25 - 28 km/h, on resistance level 4
Cool down: 2 min, at 16 km/h, on resistance level 1

**Scheduled Exercise**	(Circle answer)
Did you complete both parts of today's exercise program as indicated?	Yes / No
**If NO:**	(Circle answer)
Did you partially complete the program?	Yes / No
Please describe the exercise you managed, on the exercycle:<br>• Length of exercise time:<br>• Training km/h:<br>• Resistance setting:	Time:<br><br>km/h:<br><br>Resistance:
Did you not complete your set exercise program because you were injured or unwell?	Yes / No
If so, please briefly describe any illness or injuries that impacted your ability to exercise:	

**Un-Scheduled Exercise**<br>Exercise performed in addition to the provided study program
Please briefly list the type of additional exercise performed and time spent.

Week 7, Day 4 Weekday & Date: ________________

Rest Day

Un-Scheduled Exercise<br>Exercise performed in addition to the provided study program
Please briefly list the type of additional exercise performed and time spent.

Week 7, Day 5 Weekday & Date: ________________

Today's Exercise Schedule

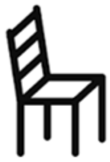

Part 1: Chair stands

Perform the chair stand by sitting and standing from a chair as many times as possible for 30 seconds.

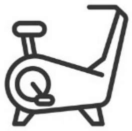

Part 2: Exercycle

Warm-up: 2 min, at 17 km/h, on resistance level 1
Training: 25 min, at 25 – 28 km/h, on resistance level 4
Cool down: 2 min, at 16 km/h, on resistance level 1

**Scheduled Exercise**	(Circle answer)
Did you complete in the 2 parts of today's exercise program as indicated?	Yes / No
**If NO:**	Circle answer)
Did you partially complete the program?	Yes / No
Please describe the exercise you managed, on the exercycle:<br>• Length of exercise time:<br>• Training km/h:<br>• Resistance setting:	Time:<br><br>km/h:<br><br>Resistance:
Did you not complete your set exercise program because you were injured or unwell?	Yes / No
If so, please briefly describe any illness or injuries that impacted your ability to exercise:	

**Un-Scheduled Exercise**<br>Exercise performed in addition to the provided study program
Please briefly list the type of additional exercise performed and time spent.

Week 7, Day 6 Weekday & Date: ________________

Today's Dosing & Rest Day

Dosing Scheduled	(Circle answer)
Did you take 3 capsules?	Yes / No
If NO, how many did you take?	
Did you not take all or part of your treatment because you were unwell?	Yes / No
If yes, please briefly describe further:	

Un-Scheduled Exercise<br>Exercise performed in addition to the provided study program
Please briefly list the type of additional exercise performed and time spent.

Week 7, Day 7 Weekday & Date: ________________

Rest Day

Un-Scheduled Exercise<br>Exercise performed in addition to the provided study program
Please briefly list the type of additional exercise performed and time spent.

Note:

Week 8, Day 1 Weekday & Date: ______________

Today's Exercise Schedule

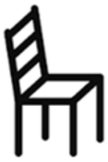

Part 1: Chair stands

Perform the chair stand by sitting and standing from a chair as many times as possible for 30 seconds.

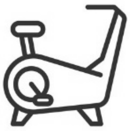

Part 2: Exercycle

Warm-up: 2 min, at 17 km/h, on resistance level 1
Training: 25 min, at 25 - 28 km/h, on resistance level 4
Cool down: 2 min, at 16 km/h, on resistance level 1

**Scheduled Exercise**	(Circle answer)
Did you complete both parts of today's exercise program as indicated?	Yes / No
**If NO:**	(Circle answer)
Did you partially complete the program?	Yes / No
Please describe the exercise you managed, on the exercycle:<br>• Length of exercise time:<br>• Training km/h:<br>• Resistance setting:	Time:<br><br>km/h:<br><br>Resistance:
Did you not complete your set exercise program because you were injured or unwell?	Yes / No
If so, please briefly describe any illness or injuries that impacted your ability to exercise:	
**Un-Scheduled Exercise**<br>Exercise performed in addition to the provided study program	
Please briefly list the type of additional exercise performed and time spent.	

Week 8, Day 2 Weekday & Date: ________________

Rest Day

Un-Scheduled Exercise<br>Exercise performed in addition to the provided study program
Please briefly list the type of additional exercise performed and time spent.

Week 8, Day 3 Weekday & Date: ________________

Today's Exercise Schedule

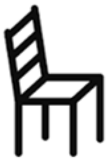

Part 1: Chair stands

Perform the chair stand by sitting and standing from a chair as many times as possible for 30 seconds.

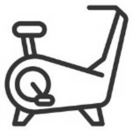

Part 2: Exercycle

Warm-up: 2 min, at 17 km/h, on resistance level 1
Training: 25 min, at 25 - 28 km/h, on resistance level 4
Cool down: 2 min, at 16 km/h, on resistance level 1

**Scheduled Exercise**	(Circle answer)
Did you complete both parts of today's exercise program as indicated?	Yes / No
**If NO:**	(Circle answer)
Did you partially complete the program?	Yes / No
Please describe the exercise you managed, on the exercycle:<br>• Length of exercise time:<br>• Training km/h:<br>• Resistance setting:	Time:<br><br>km/h:<br><br>Resistance:
Did you not complete your set exercise program because you were injured or unwell?	Yes / No
If so, please briefly describe any illness or injuries that impacted your ability to exercise:	

**Un-Scheduled Exercise**<br>Exercise performed in addition to the provided study program
Please briefly list the type of additional exercise performed and time spent.

Week 8, Day 4

Weekday & Date: ________________

Rest Day

Un-Scheduled Exercise<br>Exercise performed in addition to the provided study program
Please briefly list the type of additional exercise performed and time spent.

Week 8, Day 5 Weekday & Date: ________________

Today's Exercise Schedule

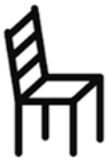

Part 1: Chair stands

Perform the chair stand by sitting and standing from a chair as many times as possible for 30 seconds.

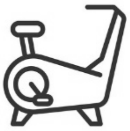

Part 2: Exercycle

Warm-up: 2 min, at 17 km/h, on resistance level 1
Training: 25 min, at 25 - 28 km/h, on resistance level 4
Cool down: 2 min, at 16 km/h, on resistance level 1

**Scheduled Exercise**	(Circle answer)
Did you complete in the 2 parts of today's exercise program as indicated?	Yes / No
**If NO:**	Circle answer)
Did you partially complete the program?	Yes / No
Please describe the exercise you managed, on the exercycle:<br>• Length of exercise time:<br>• Training km/h:<br>• Resistance setting:	Time:<br><br>km/h:<br><br>Resistance:
Did you not complete your set exercise program because you were injured or unwell?	Yes / No
If so, please briefly describe any illness or injuries that impacted your ability to exercise:	

**Un-Scheduled Exercise**<br>Exercise performed in addition to the provided study program
Please briefly list the type of additional exercise performed and time spent.

Week 8, Day 6 Weekday & Date: ________________

Today's Dosing & Rest Day

Dosing Scheduled	(Circle answer)
Did you take 3 capsules?	Yes / No
If NO, how many did you take?	
Did you not take all or part of your treatment because you were unwell?	Yes / No
If yes, please briefly describe further:	

Un-Scheduled Exercise<br>Exercise performed in addition to the provided study program
Please briefly list the type of additional exercise performed and time spent.

Week 8, Day 7 Weekday & Date: ________________

Rest Day

Un-Scheduled Exercise<br>Exercise performed in addition to the provided study program
Please briefly list the type of additional exercise performed and time spent.

Note:

Week 9, Day 1 Weekday & Date: ________________

Today's Exercise Schedule

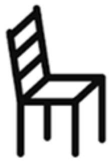

Part 1: Chair stands

Perform the chair stand by sitting and standing from a chair as many times as possible for 30 seconds.

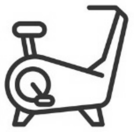

Part 2: Exercycle

Warm-up: 2 min, at 17 km/h, on resistance level 1
Training: 25 min, at 25 - 28 km/h, on resistance level 5
Cool down: 2 min, at 16 km/h, on resistance level 1

**Scheduled Exercise**	(Circle answer)
Did you complete both parts of today's exercise program as indicated?	Yes / No
**If NO:**	(Circle answer)
Did you partially complete the program?	Yes / No
Please describe the exercise you managed, on the exercycle:<br>• Length of exercise time:<br>• Training km/h:<br>• Resistance setting:	Time:<br><br>km/h:<br><br>Resistance:
Did you not complete your set exercise program because you were injured or unwell?	Yes / No
If so, please briefly describe any illness or injuries that impacted your ability to exercise:	

**Un-Scheduled Exercise**<br>Exercise performed in addition to the provided study program
Please briefly list the type of additional exercise performed and time spent.

Week 9, Day 2 Weekday & Date: ________________

Rest Day

**Un-Scheduled Exercise**<br>Exercise performed in addition to the provided study program
Please briefly list the type of additional exercise performed and time spent.

Week 9, Day 3 Weekday & Date: ________________

Today's Exercise Schedule

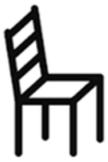

Part 1: Chair stands

Perform the chair stand by sitting and standing from a chair as many times as possible for 30 seconds.

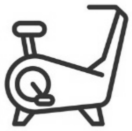

Part 2: Exercycle

Warm-up: 2 min, at 17 km/h, on resistance level 1
Training: 25 min, at 25 - 28 km/h, on resistance level 5
Cool down: 2 min, at 16 km/h, on resistance level 1

**Scheduled Exercise**	(Circle answer)
Did you complete both parts of today's exercise program as indicated?	Yes / No
**If NO:**	(Circle answer)
Did you partially complete the program?	Yes / No
Please describe the exercise you managed, on the exercycle:<br>• Length of exercise time:<br>• Training km/h:<br>• Resistance setting:	Time:<br><br>km/h:<br><br>Resistance:
Did you not complete your set exercise program because you were injured or unwell?	Yes / No
If so, please briefly describe any illness or injuries that impacted your ability to exercise:	
**Un-Scheduled Exercise**<br>Exercise performed in addition to the provided study program	
Please briefly list the type of additional exercise performed and time spent.	

Week 9, Day 4 Weekday & Date: ________________

Rest Day

**Un-Scheduled Exercise**<br>Exercise performed in addition to the provided study program
Please briefly list the type of additional exercise performed and time spent.

Week 9, Day 5 Weekday & Date: ________________

Today's Exercise Schedule

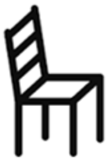

Part 1: Chair stands

Perform the chair stand by sitting and standing from a chair as many times as possible for 30 seconds.

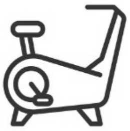

Part 2: Exercycle

Warm-up: 2 min, at 17 km/h, on resistance level 1
Training: 25 min, at 25 -28 km/h, on resistance level 5
Cool down: 2 min, at 16 km/h, on resistance level 1

**Scheduled Exercise**	(Circle answer)
Did you complete in the 2 parts of today's exercise program as indicated?	Yes / No
**If NO:**	Circle answer)
Did you partially complete the program?	Yes / No
Please describe the exercise you managed, on the exercycle:<br>• Length of exercise time:<br>• Training km/h:<br>• Resistance setting:	Time:<br><br>km/h:<br><br>Resistance:
Did you not complete your set exercise program because you were injured or unwell?	Yes / No
If so, please briefly describe any illness or injuries that impacted your ability to exercise:	
**Un-Scheduled Exercise**<br>Exercise performed in addition to the provided study program	
Please briefly list the type of additional exercise performed and time spent.	

Week 9, Day 6 Weekday & Date: ________________

Today's Dosing & Rest Day

**Dosing Scheduled**	(Circle answer)
Did you take 3 capsules?	Yes / No
If NO, how many did you take?	
Did you not take all or part of your treatment because you were unwell?	Yes / No
If yes, please briefly describe further:	

**Un-Scheduled Exercise**<br>Exercise performed in addition to the provided study program
Please briefly list the type of additional exercise performed and time spent.

Week 9, Day 7 Weekday & Date: ________________

Rest Day

**Un-Scheduled Exercise**<br>Exercise performed in addition to the provided study program
Please briefly list the type of additional exercise performed and time spent.

**Note:**

Week 10, Day 1 Weekday & Date: ________________

Today's Exercise Schedule

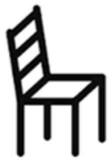

Part 1: Chair stands

Perform the chair stand by sitting and standing from a chair as many times as possible for 30 seconds.

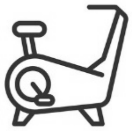

Part 2: Exercycle

Warm-up: 2 min, at 17 km/h, on resistance level 1
Training: 25 min, at 25 -28 km/h, on resistance level 5
Cool down: 2 min, at 16 km/h, on resistance level 1

**Scheduled Exercise**	(Circle answer)
Did you complete both parts of today's exercise program as indicated?	Yes / No
**If NO:**	(Circle answer)
Did you partially complete the program?	Yes / No
Please describe the exercise you managed, on the exercycle:<br>• Length of exercise time:<br>• Training km/h:<br>• Resistance setting:	Time:<br><br>km/h:<br><br>Resistance:
Did you not complete your set exercise program because you were injured or unwell?	Yes / No
If so, please briefly describe any illness or injuries that impacted your ability to exercise:	
**Un-Scheduled Exercise**<br>Exercise performed in addition to the provided study program	
Please briefly list the type of additional exercise performed and time spent.	

Week 10, Day 2 Weekday & Date: ________________

Rest Day

Un-Scheduled Exercise<br>Exercise performed in addition to the provided study program
Please briefly list the type of additional exercise performed and time spent.

Week 10, Day 3 Weekday & Date: ________________

Today's Exercise Schedule

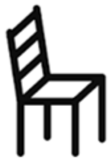

Part 1: Chair stands

Perform the chair stand by sitting and standing from a chair as many times as possible for 30 seconds.

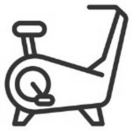

Part 2: Exercycle

Warm-up: 2 min, at 17 km/h, on resistance level 1
Training: 25 min, at 25 -28 km/h, on resistance level 5
Cool down: 2 min, at 16 km/h, on resistance level 1

**Scheduled Exercise**	(Circle answer)
Did you complete both parts of today's exercise program as indicated?	Yes / No
**If NO:**	(Circle answer)
Did you partially complete the program?	Yes / No
Please describe the exercise you managed, on the exercycle:<br>• Length of exercise time:<br>• Training km/h:<br>• Resistance setting:	Time:<br><br>km/h:<br><br>Resistance:
Did you not complete your set exercise program because you were injured or unwell?	Yes / No
If so, please briefly describe any illness or injuries that impacted your ability to exercise:	
**Un-Scheduled Exercise**<br>Exercise performed in addition to the provided study program	
Please briefly list the type of additional exercise performed and time spent.	

Week 10, Day 4 Weekday & Date: ________________

Rest Day

Un-Scheduled Exercise<br>Exercise performed in addition to the provided study program
Please briefly list the type of additional exercise performed and time spent.

Week 10, Day 5 Weekday & Date: ________________

Today's Exercise Schedule

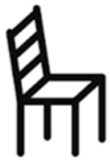

Part 1: Chair stands

Perform the chair stand by sitting and standing from a chair as many times as possible for 30 seconds.

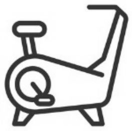

Part 2: Exercycle

Warm-up: 2 min, at 17 km/h, on resistance level 1
Training: 25 min, at 25 - 28 km/h, on resistance level 5
Cool down: 2 min, at 16 km/h, on resistance level 1

**Scheduled Exercise**	(Circle answer)
Did you complete in the 2 parts of today's exercise program as indicated?	Yes / No
**If NO:**	Circle answer)
Did you partially complete the program?	Yes / No
Please describe the exercise you managed, on the exercycle:<br>• Length of exercise time:<br>• Training km/h:<br>• Resistance setting:	Time:<br><br>km/h:<br><br>Resistance:
Did you not complete your set exercise program because you were injured or unwell?	Yes / No
If so, please briefly describe any illness or injuries that impacted your ability to exercise:	
**Un-Scheduled Exercise**<br>Exercise performed in addition to the provided study program	
Please briefly list the type of additional exercise performed and time spent.	

Week 10, Day 6 Weekday & Date: ________________

Today's Dosing & Rest Day

**Dosing Scheduled**	(Circle answer)
Did you take 3 capsules?	Yes / No
If NO, how many did you take?	
Did you not take all or part of your treatment because you were unwell?	Yes / No
If yes, please briefly describe further:	

**Un-Scheduled Exercise**<br>Exercise performed in addition to the provided study program
Please briefly list the type of additional exercise performed and time spent.

Week 10, Day 7 Weekday & Date: ________________

Rest Day

**Un-Scheduled Exercise**<br>Exercise performed in addition to the provided study program
Please briefly list the type of additional exercise performed and time spent.

**Note:**

Week 11, Day 1 Weekday & Date: ________________

Today's Exercise Schedule

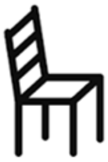

Part 1: Chair stands

Perform the chair stand by sitting and standing from a chair as many times as possible for 30 seconds.

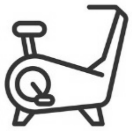

Part 2: Exercycle

Warm-up: 2 min, at 17 km/h, on resistance level 1
Training: 25 min, at 25 - 28 km/h, on resistance level 5
Cool down: 2 min, at 16 km/h, on resistance level 1

**Scheduled Exercise**	(Circle answer)
Did you complete both parts of today's exercise program as indicated?	Yes / No
**If NO:**	(Circle answer)
Did you partially complete the program?	Yes / No
Please describe the exercise you managed, on the exercycle:<br>• Length of exercise time:<br>• Training km/h:<br>• Resistance setting:	Time:<br><br>km/h:<br><br>Resistance:
Did you not complete your set exercise program because you were injured or unwell?	Yes / No
If so, please briefly describe any illness or injuries that impacted your ability to exercise:	

**Un-Scheduled Exercise**<br>Exercise performed in addition to the provided study program
Please briefly list the type of additional exercise performed and time spent.

Week 11, Day 2 Weekday & Date: ________________

Rest Day

**Un-Scheduled Exercise**<br>Exercise performed in addition to the provided study program
Please briefly list the type of additional exercise performed and time spent.

Week 11, Day 3 Weekday & Date: ________________

Today's Exercise Schedule

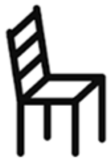

Part 1: Chair stands

Perform the chair stand by sitting and standing from a chair as many times as possible for 30 seconds.

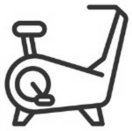

Part 2: Exercycle

Warm-up: 2 min, at 17 km/h, on resistance level 1
Training: 25 min, at 25 - 28 km/h, on resistance level 5
Cool down: 2 min, at 16 km/h, on resistance level 1

**Scheduled Exercise**	(Circle answer)
Did you complete both parts of today's exercise program as indicated?	Yes / No
**If NO:**	(Circle answer)
Did you partially complete the program?	Yes / No
Please describe the exercise you managed, on the exercycle:<br>• Length of exercise time:<br>• Training km/h:<br>• Resistance setting:	Time:<br><br>km/h:<br><br>Resistance:
Did you not complete your set exercise program because you were injured or unwell?	Yes / No
If so, please briefly describe any illness or injuries that impacted your ability to exercise:	

**Un-Scheduled Exercise**<br>Exercise performed in addition to the provided study program
Please briefly list the type of additional exercise performed and time spent.

Week 11, Day 4 Weekday & Date: ________________

Rest Day

**Un-Scheduled Exercise**<br>Exercise performed in addition to the provided study program
Please briefly list the type of additional exercise performed and time spent.

Week 11, Day 5 Weekday & Date: ________________

Today's Exercise Schedule

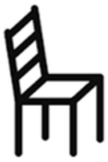

Part 1: Chair stands

Perform the chair stand by sitting and standing from a chair as many times as possible for 30 seconds.

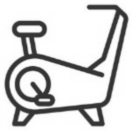

Part 2: Exercycle

Warm-up: 2 min, at 17 km/h, on resistance level 1
Training: 25 min, at 25 - 28 km/h, on resistance level 5
Cool down: 2 min, at 16 km/h, on resistance level 1

**Scheduled Exercise**	(Circle answer)
Did you complete in the 2 parts of today's exercise program as indicated?	Yes / No
**If NO:**	Circle answer)
Did you partially complete the program?	Yes / No
Please describe the exercise you managed, on the exercycle:<br>• Length of exercise time:<br>• Training km/h:<br>• Resistance setting:	Time:<br><br>km/h:<br><br>Resistance:
Did you not complete your set exercise program because you were injured or unwell?	Yes / No
If so, please briefly describe any illness or injuries that impacted your ability to exercise:	
**Un-Scheduled Exercise**<br>Exercise performed in addition to the provided study program	
Please briefly list the type of additional exercise performed and time spent.	

Week 11, Day 6 Weekday & Date: ________________

Today's Dosing & Rest Day

**Dosing Scheduled**	(Circle answer)
Did you take 3 capsules?	Yes / No
If NO, how many did you take?	
Did you not take all or part of your treatment because you were unwell?	Yes / No
If yes, please briefly describe further:	

**Un-Scheduled Exercise**<br>Exercise performed in addition to the provided study program
Please briefly list the type of additional exercise performed and time spent.

Week 11, Day 7 Weekday & Date: ________________

Rest Day

**Un-Scheduled Exercise**<br>Exercise performed in addition to the provided study program
Please briefly list the type of additional exercise performed and time spent.

**Note:**

Week 12, Day 1 Weekday & Date: ________________

Today's Exercise Schedule

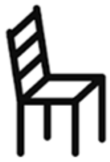

Part 1: Chair stands

Perform the chair stand by sitting and standing from a chair as many times as possible for 30 seconds.

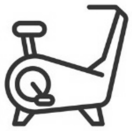

Part 2: Exercycle

Warm-up: 2 min, at 17 km/h, on resistance level 1
Training: 25 min, at 25 - 28 km/h, on resistance level 5
Cool down: 2 min, at 16 km/h, on resistance level 1

**Scheduled Exercise**	(Circle answer)
Did you complete both parts of today's exercise program as indicated?	Yes / No
**If NO:**	(Circle answer)
Did you partially complete the program?	Yes / No
Please describe the exercise you managed, on the exercycle:<br>• Length of exercise time:<br>• Training km/h:<br>• Resistance setting:	Time:<br><br>km/h:<br><br>Resistance:
Did you not complete your set exercise program because you were injured or unwell?	Yes / No
If so, please briefly describe any illness or injuries that impacted your ability to exercise:	
**Un-Scheduled Exercise**<br>Exercise performed in addition to the provided study program	
Please briefly list the type of additional exercise performed and time spent.	

Week 12, Day 2 Weekday & Date: ________________

Rest Day

**Un-Scheduled Exercise**<br>Exercise performed in addition to the provided study program
Please briefly list the type of additional exercise performed and time spent.

Week 12, Day 3 Weekday & Date: ________________

Today's Exercise Schedule

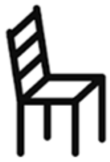

Part 1: Chair stands

Perform the chair stand by sitting and standing from a chair as many times as possible for 30 seconds.

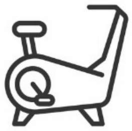

Part 2: Exercycle

Warm-up: 2 min, at 17 km/h, on resistance level 1
Training: 25 min, at 25 - 28 km/h, on resistance level 5
Cool down: 2 min, at 16 km/h, on resistance level 1

**Scheduled Exercise**	(Circle answer)
Did you complete both parts of today's exercise program as indicated?	Yes / No
**If NO:**	(Circle answer)
Did you partially complete the program?	Yes / No
Please describe the exercise you managed, on the exercycle:<br>• Length of exercise time:<br>• Training km/h:<br>• Resistance setting:	Time:<br><br>km/h:<br><br>Resistance:
Did you not complete your set exercise program because you were injured or unwell?	Yes / No
If so, please briefly describe any illness or injuries that impacted your ability to exercise:	

**Un-Scheduled Exercise**<br>Exercise performed in addition to the provided study program
Please briefly list the type of additional exercise performed and time spent.

Week 12, Day 4 Weekday & Date: ________________

Rest Day

Un-Scheduled Exercise<br>Exercise performed in addition to the provided study program
Please briefly list the type of additional exercise performed and time spent.

Week 12, Day 5 Weekday & Date: ________________

Today's Exercise Schedule

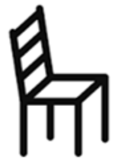

Part 1: Chair stands

Perform the chair stand by sitting and standing from a chair as many times as possible for 30 seconds.

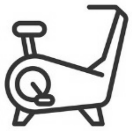

Part 2: Exercycle

Warm-up: 2 min, at 17 km/h, on resistance level 1
Training: 25 min, at 25 - 28 km/h, on resistance level 5
Cool down: 2 min, at 16 km/h, on resistance level 1

**Scheduled Exercise**	(Circle answer)
Did you complete in the 2 parts of today's exercise program as indicated?	Yes / No
**If NO:**	Circle answer)
Did you partially complete the program?	Yes / No
Please describe the exercise you managed, on the exercycle:<br>• Length of exercise time:<br>• Training km/h:<br>• Resistance setting:	Time:<br><br>km/h:<br><br>Resistance:
Did you not complete your set exercise program because you were injured or unwell?	Yes / No
If so, please briefly describe any illness or injuries that impacted your ability to exercise:	

**Un-Scheduled Exercise**<br>Exercise performed in addition to the provided study program
Please briefly list the type of additional exercise performed and time spent.

Week 12, Day 6 Weekday & Date: ________________

Today's Dosing & Rest Day

**Dosing Scheduled**	(Circle answer)
Did you take 3 capsules?	Yes / No
If NO, how many did you take?	
Did you not take all or part of your treatment because you were unwell?	Yes / No
If yes, please briefly describe further:	

**Un-Scheduled Exercise**<br>Exercise performed in addition to the provided study program
Please briefly list the type of additional exercise performed and time spent.

Week 12, Day 7 Weekday & Date: ________________

Rest Day

**Un-Scheduled Exercise**<br>Exercise performed in addition to the provided study program
Please briefly list the type of additional exercise performed and time spent.

**Note:**

Week 13, Day 1 Weekday & Date: ________________

Today's Exercise Schedule

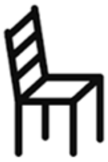

Part 1: Chair stands

Perform the chair stand by sitting and standing from a chair as many times as possible for 30 seconds.

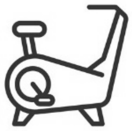

Part 2: Exercycle

Warm-up: 2 min, at 17 km/h, on resistance level 1
Training: 25 min, at 25 -28 km/h, on resistance level 5
Cool down: 2 min, at 16 km/h, on resistance level 1

**Scheduled Exercise**	(Circle answer)
Did you complete both parts of today's exercise program as indicated?	Yes / No
**If NO:**	(Circle answer)
Did you partially complete the program?	Yes / No
Please describe the exercise you managed, on the exercycle:<br>• Length of exercise time:<br>• Training km/h:<br>• Resistance setting:	Time:<br><br>km/h:<br><br>Resistance:
Did you not complete your set exercise program because you were injured or unwell?	Yes / No
If so, please briefly describe any illness or injuries that impacted your ability to exercise:	

**Un-Scheduled Exercise**<br>Exercise performed in addition to the provided study program
Please briefly list the type of additional exercise performed and time spent.

Week 13, Day 2 Weekday & Date: ________________

Rest Day

Un-Scheduled Exercise<br>Exercise performed in addition to the provided study program
Please briefly list the type of additional exercise performed and time spent.

Week 13, Day 3 Weekday & Date: ________________

Today's Exercise Schedule

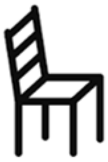

Part 1: Chair stands

Perform the chair stand by sitting and standing from a chair as many times as possible for 30 seconds.

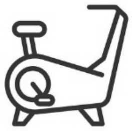

Part 2: Exercycle

Warm-up: 2 min, at 17 km/h, on resistance level 1
Training: 25 min, at 25 -28 km/h, on resistance level 5
Cool down: 2 min, at 16 km/h, on resistance level 1

**Scheduled Exercise**	(Circle answer)
Did you complete both parts of today's exercise program as indicated?	Yes / No
**If NO:**	(Circle answer)
Did you partially complete the program?	Yes / No
Please describe the exercise you managed, on the exercycle:<br>• Length of exercise time:<br>• Training km/h:<br>• Resistance setting:	Time:<br><br>km/h:<br><br>Resistance:
Did you not complete your set exercise program because you were injured or unwell?	Yes / No
If so, please briefly describe any illness or injuries that impacted your ability to exercise:	
**Un-Scheduled Exercise**<br>Exercise performed in addition to the provided study program	
Please briefly list the type of additional exercise performed and time spent.	

Week 13, Day 4 Weekday & Date: ________________

Rest Day

**Un-Scheduled Exercise**<br>Exercise performed in addition to the provided study program
Please briefly list the type of additional exercise performed and time spent.

Week 13, Day 5 Weekday & Date: ________________

Today's Exercise Schedule

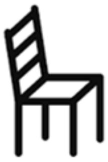

Part 1: Chair stands

Perform the chair stand by sitting and standing from a chair as many times as possible for 30 seconds.

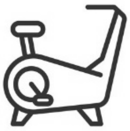

Part 2: Exercycle

Warm-up: 2 min, at 17 km/h, on resistance level 1
Training: 25 min, at 25 - 28 km/h, on resistance level 5
Cool down: 2 min, at 16 km/h, on resistance level 1

**Scheduled Exercise**	(Circle answer)
Did you complete in the 2 parts of today's exercise program as indicated?	Yes / No
**If NO:**	Circle answer)
Did you partially complete the program?	Yes / No
Please describe the exercise you managed, on the exercycle:<br>• Length of exercise time:<br>• Training km/h:<br>• Resistance setting:	Time:<br><br>km/h:<br><br>Resistance:
Did you not complete your set exercise program because you were injured or unwell?	Yes / No
If so, please briefly describe any illness or injuries that impacted your ability to exercise:	
**Un-Scheduled Exercise**<br>Exercise performed in addition to the provided study program	
Please briefly list the type of additional exercise performed and time spent.	

Week 13, Day 6 Weekday & Date: ________________

Today's Dosing & Rest Day

**Dosing Scheduled**	(Circle answer)
Did you take 3 capsules?	Yes / No
If NO, how many did you take?	
Did you not take all or part of your treatment because you were unwell?	Yes / No
If yes, please briefly describe further:	

**Un-Scheduled Exercise**<br>Exercise performed in addition to the provided study program
Please briefly list the type of additional exercise performed and time spent.

Week 13, Day 7 Weekday & Date: ________________

Rest Day

**Un-Scheduled Exercise**<br>Exercise performed in addition to the provided study program
Please briefly list the type of additional exercise performed and time spent.

**Note:**
